# Molecular basis for the regulation of human glycogen synthase by phosphorylation and glucose-6-phosphate

**DOI:** 10.1038/s41594-022-00799-3

**Published:** 2022-07-14

**Authors:** Thomas J. McCorvie, Paula M. Loria, Meihua Tu, Seungil Han, Leela Shrestha, D. Sean Froese, Igor M. Ferreira, Allison P. Berg, Wyatt W. Yue

**Affiliations:** 1 Centre for Medicines Discovery, Nuffield Department of Clinical Medicine, University of Oxford, Oxford, UK; 2 Discovery Sciences, Worldwide Research and Development, Pfizer Inc., Groton, CT USA; 3 Medicine Design, Worldwide Research and Development, Pfizer Inc., Cambridge, MA USA; 4 Rare Disease Research Unit, Worldwide Research and Development, Pfizer Inc., Cambridge, MA USA; 5 Present Address: Biosciences Institute, The Medical School, Newcastle University, Newcastle upon Tyne, UK; 6 Present Address: Division of Metabolism and Children’s Research Center, University Children’s Hospital Zürich, University of Zürich, Zürich, Switzerland

**Keywords:** Cryoelectron microscopy, Transferases, Carbohydrates

## Abstract

Glycogen synthase (GYS1) is the central enzyme in muscle glycogen biosynthesis. GYS1 activity is inhibited by phosphorylation of its amino (N) and carboxyl (C) termini, which is relieved by allosteric activation of glucose-6-phosphate (Glc6P). We present cryo-EM structures at 3.0–4.0 Å resolution of phosphorylated human GYS1, in complex with a minimal interacting region of glycogenin, in the inhibited, activated and catalytically competent states. Phosphorylations of specific terminal residues are sensed by different arginine clusters, locking the GYS1 tetramer in an inhibited state via intersubunit interactions. The Glc6P activator promotes conformational change by disrupting these interactions and increases the flexibility of GYS1, such that it is poised to adopt a catalytically competent state when the sugar donor UDP-glucose (UDP-glc) binds. We also identify an inhibited-like conformation that has not transitioned into the activated state, in which the locking interaction of phosphorylation with the arginine cluster impedes subsequent conformational changes due to Glc6P binding. Our results address longstanding questions regarding the mechanism of human GYS1 regulation.

## Abstract (web summary)

Multiple cryo-EM structures of human glycogen synthase reveal the basis of inhibition by phosphorylation and allosteric activation by glucose-6-phosphate.

## Main

**Fig. 1:**
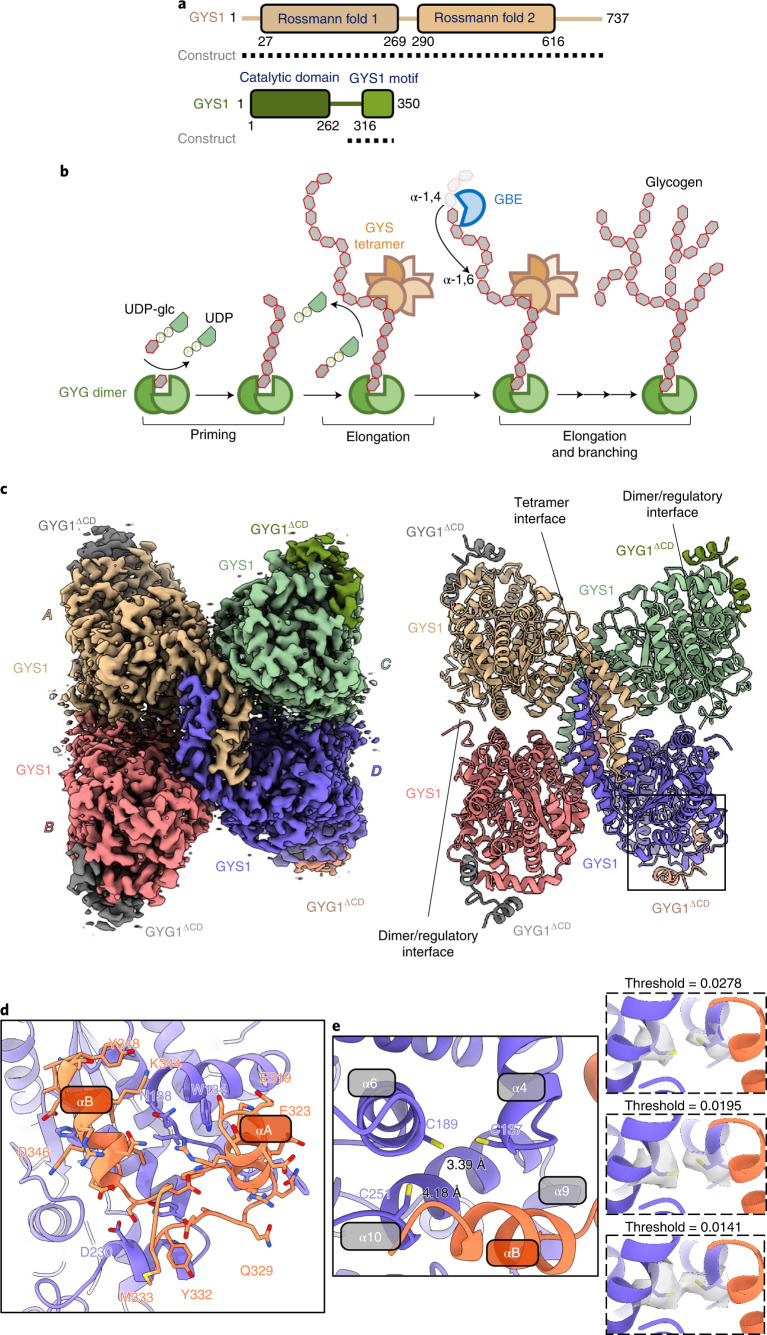
Structure of the phosphorylated inhibited (T state) GYS1–GYG1^ΔCD^ complex. **a**, Domain diagrams of human GYS1 and GYG1. Dotted lines represent the construct boundaries of the GYS1–GYG1^ΔCD^ complex used in all cryo-EM experiments. **b**, Schematic of the enzyme-catalyzed reactions of GYG1, GYS1 and GBE. Glycogen synthesis is a multistep process consisting of a priming step by GYG followed by an elongation step carried out by GYS and then a branching step by GBE. **c**, Cryo-EM map and model of the tetrameric GYS1–GYG1^ΔCD^ complex at 3.0 Å resolution. Individual GYS1 and GYG1 subunits are coloured separately. **d**, Enlarged view of the GYG1 region interacting with GYS1. GYS1 is coloured purple and GYG1 is coloured coral. **e**, Residues Cys137, Cys189 and Cys251 form a cysteine-rich pocket on GYS1 at the interface with GYG1. Inset shows different contour levels for the cryo-EM density of Cys137 and Cys189.

Glycogen serves as the main carbohydrate and energy reserve across animal phyla, containing more than 55,000 glucose units linked by α-1,4 and α-1,6 glucosidic bonds^1^. Glycogen biosynthesis is catalyzed by three enzymes in eukaryotes: (1) glycogenin (GYG, EC 2.4.1.186), which forms a short primer through stepwise attachment of glucose units onto itself^2^; (2) glycogen synthase (GYS, EC 2.4.1.11), which ‘strings’ glucose units to elongate the GYG-attached primer^3^; and (3) glycogen branching enzyme (GBE, EC 2.4.1.18), which introduces branch points to a linear chain via α-1,6 linkages^4^ (Fig. 1b).

GYS, a retaining glycosyltransferase (GT) belonging to the GT3 superfamily, catalyzes successive addition of α-1,4-linked glucose residues to the nonreducing end of a growing polysaccharide chain, using UDP-glc as the sugar donor with the release of UDP^5^. Mammalian GYS comprises two isoforms, GYS1 and GYS2, with ~69% sequence identity^6^. GYS1 is expressed in most tissues including the muscle and brain^7^, whereas GYS2 is expressed only in the liver. Mammalian GYS is the rate-limiting enzyme in glycogen biosynthesis, and its activity is regulated posttranslationally by two mechanisms: activation by the effector Glc6P^8,9^ and inhibition by reversible phosphorylation^10^.

Reversible phosphorylation of GYS, mediated by Ser/Thr-directed protein kinases, occurs at multiple sites and is hierarchal; that is, different sites contribute to GYS inhibition in a specific order and to varying degrees^11^. At least nine phosphorylation sites have been identified in vivo at the N and C termini of mammalian GYS1, of which sites 2 (Ser8), 2a (Ser11), 3a (Ser641) and 3b (Ser645) have the most substantial roles^12,13^. Dephosphorylation, performed by glycogen-associated phosphatases of type 1 (PP1), substantially alters kinetic properties of GYS, including increased affinity for UDP-glc and sensitivity to the Glc6P activator^14^. Glc6P binds to an allosteric site equipped with an arginine cluster, overcomes phosphorylation-dependent inhibition, and increases the enzyme’s susceptibility to PP1-mediated dephosphorylation. The two regulatory mechanisms of mammalian GYS follow a three-state conformational model, comprising the tense (T) or inhibited state in which GYS is phosphorylated, the intermediate (I) or basal state in which it is unphosphorylated and the relaxed (R) or activated state in which Glc6P is bound^15–18^.

The pleiotropic PP1 comprises a catalytic subunit (PP1c) and a regulatory subunit (PP1r), with the latter targeting the phosphatase to specific targets. Seven glycogen-targeting PP1r subunits (PPP1R3A to PPP1R3G) have been described, each comprising an RVSF motif for PP1c binding, a glycogen-binding motif VxNxxFEKxV and a putative GYS-binding motif WxNxGxNYx(I/L)^19–21^. Subunit PPP1R3C (also known as protein targeting to glycogen, PTG) is ubiquitously expressed in the brain, liver and heart, and its gene knockout indirectly reduces GYS activation^22^. These PP1 regulatory subunits are often considered to be activators of GYS1, and PTG is thought to function as a scaffold for glycogen metabolic enzymes such as GYS, glycogen phosphorylase and phosphorylase kinase^22^.

GYS1 has emerged as a therapeutic target for several glycogen storage diseases (GSD), including GSD type II (Pompe disease)^23^, GSD type IV (Andersen disease and adult polyglucosan body disease)^24^ and Lafora disease^25^. The root of these disorders is the accumulation of aberrant or normal glycogen in affected tissues, due to defective glycogen synthesis or breakdown. Downregulation of GYS1 activity to interfere with glycogen chain elongation has therapeutic potential. Despite this, inhibitor development for GYS1 has not progressed rapidly^26,27^, partly owing to a lack of GYS structures other than those from bacteria^28–30^, *Saccharomyces cerevisiae*^16^ and *Caenorhabditis* *elegans*^31^ to guide drug discovery efforts. Here, we determined a cryo-EM structure of phosphorylated human GYS1 in different functional states and characterized its interactions with its functional partners, glycogenin GYG1 and PTG.

## Results

### Structure of human GYS1 with interacting region of GYG1

**Extended Data Fig. 1:**
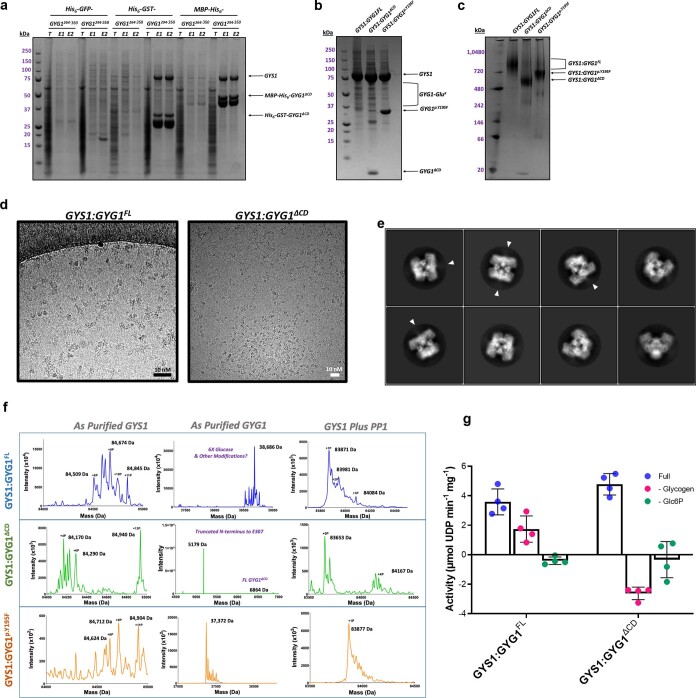
Purification and preliminary characterization of GYS1:GYG1 complexes. **a**, Coomassie stained SDS-PAGE of small-scale test purifications of GYS1 complexed with differently tagged truncated GYG1. **b**, Coomassie stained SDS-PAGE of the three GYS1:GYG1 complexes used in this study. **c**, Blue native PAGE of the three GYS1:GYG1 complexes used in this study. **d**, Example micrographs of GYS1:GYG1^FL^ and GYS1:GYG1^ΔCD^ complexes collected using a Glacios microscope. **e**, 2D classes of the GYS1:GYG1^ΔCD^ complex from an initial dataset collected using a Glacios microscope. Arrows indicate regions of fuzzy density protruding from an inter-subunit interface. **f**, Denaturing mass-spectra of GYS1 and GYG1, as purified and treated with PP1. **g**, UDP-Glo activity assay of the three GYS1:GYG1 constructs without and with exogenous glycogen. ‘Full’ is the activity assay with all substrates. ‘- Glycogen’ is the assay carried out in the absence of exogenously added glycogen. ‘- Glc6P’ is the assay carried out in the absence of Glc6P. Median and standard deviation of activity is shown (n = 3 technical repeats). Source data

Unlike *C. elegans* gsy-1 and yeast Gsy2p, human GYS1 has proved a challenge to produce alone in a recombinant soluble form for structural studies. However, coexpression with its binding partner, human GYG1, in an insect expression system has enabled the isolation of a ~0.5 mDa complex^32,33^. Therefore, we coexpressed and purified the full-length GYS1–GYG1^FL^ complex (Extended Data Fig. 1b) but found it to be recalcitrant for crystallization. This was probably because of a combination of flexible regions along with heterogeneous phosphorylation and glucosylation of GYS1 and GYG1, as reported previously^32,33^ and determined by denaturing mass spectrometry (Extended Data Fig. 1f). The GYS1–GYG1^FL^ complex was prone to aggregation and showed heterogenous particle sizes (Extended Data Fig. 1d) in cryo-EM.

Human GYG1 comprises the N-terminal catalytic domain, a flexible linker and the C-terminal GYS1-interacting domain (Fig. 1a). The crystal structure of full-length *C. elegans* gsy-1 in complex with the last 34 residues of glycogenin (gyg-1) demonstrated that this highly conserved gyg-1 C terminus forms a helix-turn-helix motif sufficient for interaction with GYS1 (ref. ^31^). In our attempts to improve the complex for crystallization, we designed bicistronic constructs encoding untagged human GYS1 (amino acids (aa) 1–737) and the His_6_-GST-tagged GYG1 C terminus (aa 264–350 or aa 294–350). Coexpression with GYG1 294–350 produced soluble GYS1 (Extended Data Fig. 1a). This construct (GYS1–GYG1^ΔCD^) was multiply phosphorylated, as determined by intact mass spectrometry (Extended Data Fig. 1f). This truncated complex had similar GT activity to that of the wild-type GYS1–GYG1^FL^ complex, and likewise it was stimulated by Glc6P (Extended Data Fig. 1g). Despite considerable effort, no crystals of GYS1–GYG1^ΔCD^ were obtained; however, it presented less aggregation than GYS1–GYG1^FL^ in cryo-EM grids. Individual box-shaped particles were discernible and initial two-dimensional (2D) classification resulted in classes representative of a tetrameric particle (Extended Data Fig. 1d,e).

**Extended Data Fig. 2:**
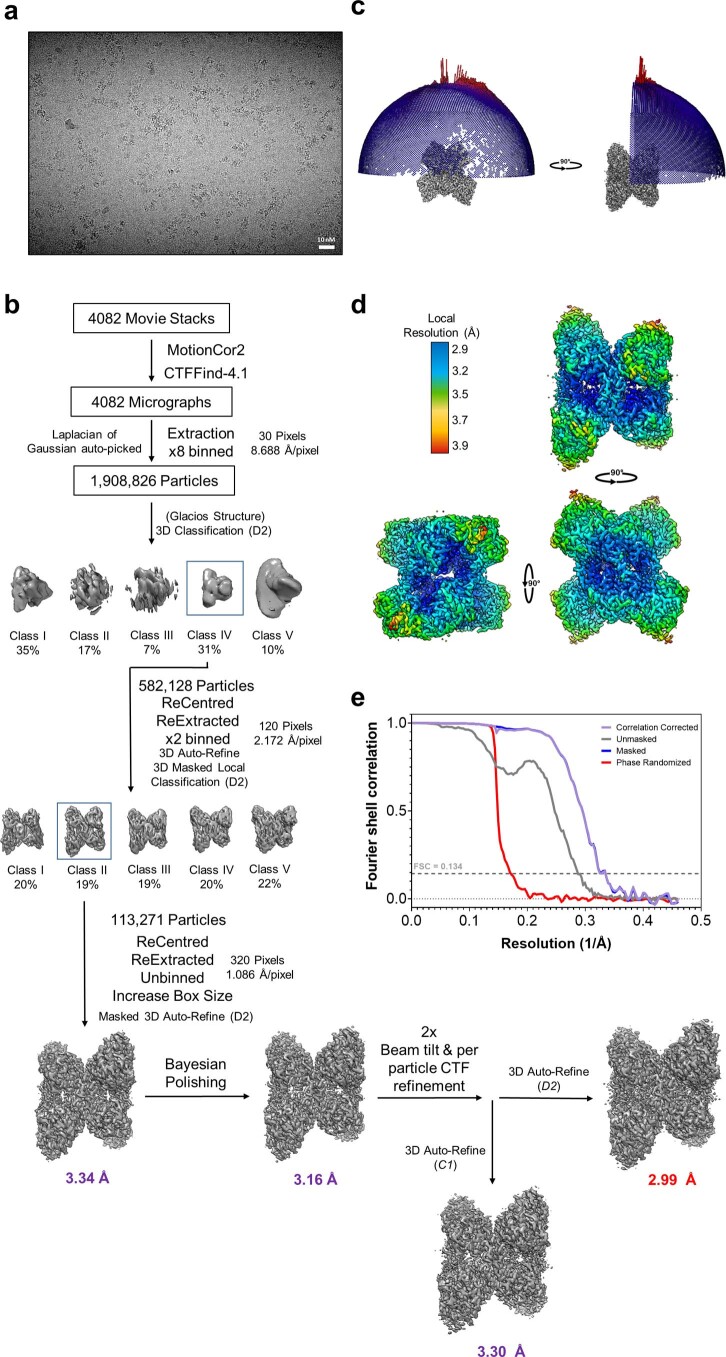
Image processing workflow of the GYS1:GYG1^ΔCD^ inhibited state. **a**, Representative K3 micrograph of the GYS1:GYG1^ΔCD^ inhibited state from 4082 micrographs collected. **b**, Processing flow chart of the GYS1 + GYG1^ΔCD^ inhibited state. **c**, Angular distribution of the 3.0 Å GYS1:GYG1^ΔCD^ inhibited state map. **d**, Local resolution variation of the 3.0 Å GYS1:GYG1^ΔCD^ inhibited state map. **e**, FSC curve of the 3.0 Å GYS1:GYG1^ΔCD^ inhibited state map.

**Extended Data Fig. 3:**
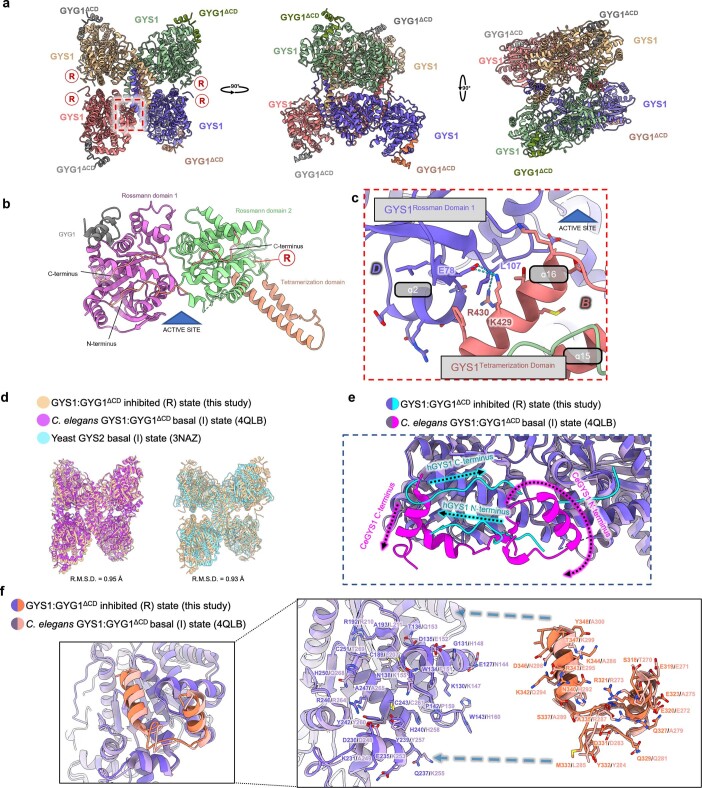
Structure of the GYS1:GYG1^ΔCD^ inhibited state and comparison with the *C. elegans* gsy-1 and yeast Gsy2p basal/intermediate state structures. **a**, Model of the GYS1:GYG1^ΔCD^ inhibited state in three orthogonal views. R represents the location of the regulatory helix. **b**, Structural model of a GYS1:GYG1^ΔCD^ subunit showing the three domains of GYS1 as well as the GYG1 C-terminus. **c**, Close up of the inter-subunit interactions close to the active site cleft. **d**, Structural alignment of the inhibited/T state of the human GYS1:GYG1^ΔCD^ complex with the basal/I states of yeast Gsy2p and *C. elegans* gsy-1:gyg-1^ΔCD^ complex. **e**, A zoom in view of the GYG1 interacting region of GYS1 of human and *C. elegans*. **f**, A structural alignment of the inhibited/T state of human GYS1 against the basal/I state of *C. elegans* gsy-1 highlighting the different trajectories of the N- and C- termini.

We determined a 3.0 Å structure of a phosphorylated GYS1–GYG1^ΔCD^ complex with *D2* symmetry applied (Fig. 1c, Table 1, and Extended Data Fig. 2). The cryo-EM map ranged from 2.9 Å resolution at the core to 3.9 Å resolution at the periphery of the complex, allowing for modelling of residues 13–289, 293–629 and 637–645 of GYS1 and residues 317–349 of GYG1. The complex adopted a rectangular box shape, with residues 317–349 of GYG1 at each corner of the GYS1 homotetramer (Fig. 1c and Extended Data Fig. 3a). Each GYS1 monomer consisted of two Rossmann domains and a tetramerization domain and interacted with GYG1 in a 1:1 ratio (Extended Data Fig. 3b). GYS1 assembled into a dimer of dimers with two major interfaces (Fig. 1c and Extended Data Fig. 3a): a tetrameric interface formed by tetramerization domains (A–D and B–C interfaces) and a dimeric regulatory interface (C–D and A–B interfaces). The latter was contributed by the regulatory helix α24 from each subunit, harboring conserved arginine clusters. In this structure, each GYS1 active site, at the cleft between the two Rossmann domains, was in a closed conformation owing to additional intersubunit contacts at a minor interface (B–D or A–C)^16,31^. Here, helix α2 of Rossmann domain 1 contacted helix α16 of the tetramerization domain of the neighbouring subunit via a salt bridge between Glu78 and Lys429 along with a hydrogen bond between Leu107 and Arg430 (Extended Data Fig. 3c).

**Table 1:** Cryo-EM data collection, refinement and validation statistics

	Inhibited state (EMDB-13743) (PDB 7Q0B)	+Glc6P, inhibited-like state (EMDB-13751) (PDB 7Q0S)	+Glc6P, activated state (EMDB-13752) (PDB 7Q12)	+Glc6P +UDP-glc, activated state (EMDB-13753) (PDB 7Q13)
**Data collection and processing**				
Magnification	81,000	81,000		81,000
Voltage (kV)	300	300		300
Electron exposure (e^–^/Å^2^)	55.0	55.0		50.00
Defocus range (μm)	−0.8 to −2.3	−0.8 to −2.3		−0.8 to −2.3
Pixel size (Å)	1.086	1.086		1.06
Symmetry imposed	*D*2	*D*2	*D*2	*D*2
Initial particle images (no.)	1,908,826	4,391,867	4,391,867	10,011,868
Final particle images (no.)	113,271	40,062	15,379	35,604
Map resolution (Å)	3.0	4.0	3.7	3.0
FSC threshold	0.143	0.143	0.143	0.143
Map resolution range (Å)	2.9–3.9	3.6–6.2	3.6–6.4	2.8–4.9
**Refinement**				
Initial model used (PDB code)	4QLB	4QLB	3NB0, 4QLB	3NB0, 4QLB
Model resolution (Å)	3.1	4.1	3.7	3.1
FSC threshold	0.5	0.5	0.5	0.5
Model resolution range (Å)				
Map-sharpening *B* factor (Å^2^)	−68	−143	−95	−51
Model composition				
Nonhydrogen atoms	21,172	21,196	20,240	20,372
Protein residues	2,618	2,612	2,488	2,488
Ligands	0	4 G6P	4 G6P	4 G6P, 4 GLC, 4 UDP
*B* factors (Å^2^)				
Protein	29.87	81.36	116.12	41.65
Ligand		54.43	32.41	25.15
R.m.s. deviations				
Bond lengths (Å)	0.004	0.003	0.002	0.003
Bond angles (°)	0.542	0.592	0.535	0.589
**Validation**				
MolProbity score	1.48	1.58	1.67	1.93
Clashscore	4.77	9.02	8.33	11.38
Poor rotamers (%)	0.13	0.00	0.00	0.37
Ramachandran plot				
Favoured (%)	96.43	97.52	96.64	94.78
Allowed (%)	3.57	2.48	3.56	5.22
Disallowed (%)	0.00	0.00	0.00	0.00

The interactions of GYG1 with GYS1 were similar to that found in the *C. elegans* crystal structure^31^ (Fig. 1d and Extended Data Fig. 3f). GYG1 used a helix (αA)-turn-helix (αB) motif to interact with helices α4, α9 and α10 of GYS1, through hydrogen bonds and hydrophobic interactions (Fig. 1d and Extended Data Fig. 3f). At the GYS1 region where GYG1 interacted, we observed a cysteine-rich pocket of residues, Cys137, Cys189 and Cys251, near the last α-helix of GYG1 (Fig. 1e). The distances between Cys137 and Cys189 (3.39 Å) and between Cys189 and Cys251 (4.18 Å) were within disulfide-bonding distance. Lower threshold values of the cryo-EM density suggested a possible disulfide bond between Cys137 and Cys189 (Fig. 1e inset); however, owing to the ambiguity we modelled all three cysteine residues as reduced. Without GYG1, the GYS1 cysteine-rich pocket would be solvent-exposed; thus, GYG1 may stabilize this region by preventing aberrant disulfide formation. The lack of this cysteine-rich pocket (Cys137, Cys189, Cys251) in yeast Gsy2p (Val126, Pro177, Ser240) and *C. elegans* gsy-1 (Cys154, Leu207, Thr269) may explain the unique requirement of coexpressing GYG1 to stabilize human GYS1 (Supplementary Fig. 1). One might speculate that these cysteines act as a redox switch, as found in human brain glycogen phosphorylase^34^; this possibility should be investigated in future studies.

### Structural basis of phosphorylation sensing

**Fig. 2:**
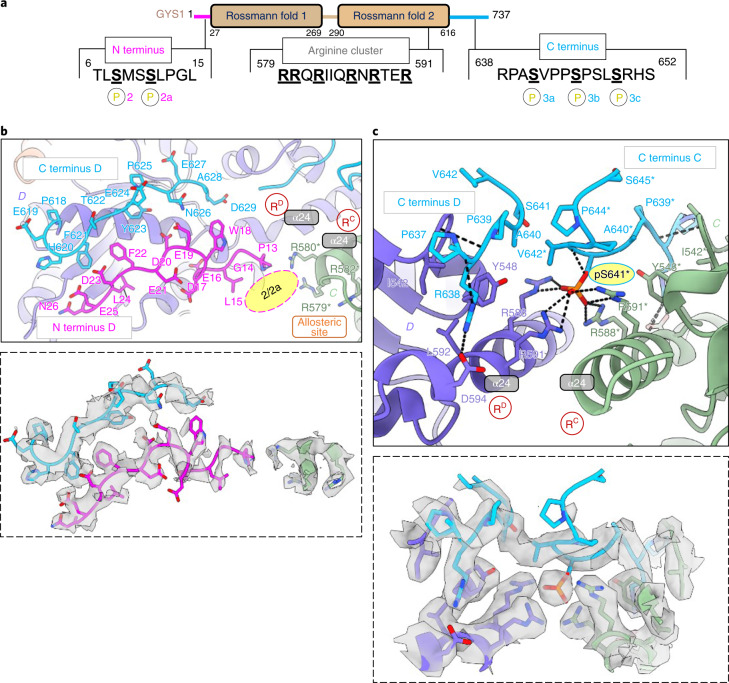
N and C termini of phosphorylated GYS1–GYG1^ΔCD^ complex in the inhibited (T) state. **a**, Key sites of phosphorylation and arginine cluster of a GYS1 subunit. **b**, Model of the N and C termini of one subunit (D shown) pointing towards the allosteric sites and arginine clusters (R^C^ and R^D^) at the dimeric C–D interface. Inset shows the EM density of both termini, along with arginine residues from the neighbouring subunit that would interact with phosphorylation sites 2 and 2a. **c**, Model of C-terminal residues 637–645 from two neighbouring subunits (C and D shown) interacting with their arginine clusters at the dimeric C–D interface. Inset shows EM density of both C termini along with arginine clusters from both subunits interacting with a single site 3a phosphorylation (pS641). Asterisks indicate residues from the neighbouring subunit. Arginine clusters containing α24 helices are labelled. Putative locations for phosphorylation sites 2 and 2a are indicated by pink ovals.

**Extended Data Fig. 4:**
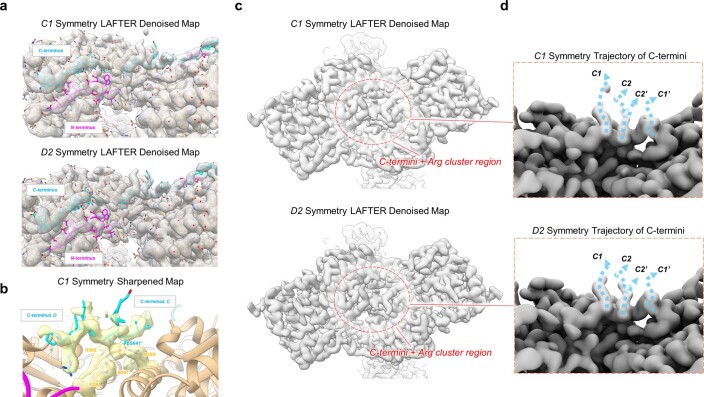
Modelling of the N- and C- termini of the inhibited/T state of the GYS1:GYG1 complex. **a**, Fitting of the N- and C- termini model into the *C1* and *D2* symmetry LAFTER denoised maps. **b**, Fitting of the phosphorylated C- termini model into the sharpened *C1* symmetry map. **c**, Views of the regulatory dimeric interface of the C1 and D2 symmetry LAFTER maps. The phosphorylated C-termini region density is symmetric in both maps. **d**, Predicted directions of the phosphorylated C-termini in C1 and D2 symmetry LAFTER denoised maps. The C-termini are predicted to continue away from the dimeric regulatory interface from two adjacent but different locations.

The as-purified GYS1 was highly phosphorylated (Extended Data Fig. 1f), representative of the T state and supported by the lack of GT activity without Glc6P (Extended Data Fig. 1g). However, GYS1 in this state adopted a similar conformation to the *C. elegans* gsy-1 (r.m.s. deviation (r.m.s.d.) 0.95 Å) and yeast Gsy2p basal (I) state (r.m.s.d. 0.93 Å) structures (Extended Data Fig. 3d). In eukaryotic GYS, the N and C termini harbor several phosphorylation sites that mediate inhibition^12,13^ (Fig. 2a), and it has been suggested that each phosphorylated site interacts with specific conserved arginine residues present on a regulatory helix α24 (refs. ^16,31^). In our 3.0 Å map, density was present for modelling the N and C termini (Fig. 2b and Extended Data Fig. 4).

Both termini followed a trajectory different from that of the nonphosphorylated *C. elegans* gsy-1 I state and did not form any secondary structure (Extended Data Fig. 3e). In the T state, the N and C termini from each subunit traversed from and toward the two regulatory α24 helices at the dimeric (C–D and A–B) interfaces, respectively. We modelled the N terminus from residue Pro13 onwards. Although there was no clear density for phosphorylation sites 2 (Ser8) and 2a (Ser11), they would be positioned near the regulatory α24 helix of the subunit across the dimeric interface, close to both Arg579 and Arg580, which could potentially sense the phosphorylation at these sites (Fig. 2b). The N and C termini from one subunit traversed in an antiparallel fashion towards its own α24 helix and the α24 helix from the subunit across the dimeric interface (Fig. 2b). In both the *C1* and *D2* symmetry maps, strong density was apparent between Arg588 and Arg591 of both GYS1 subunits at the dimeric interface (Fig. 2c and Extended Data Fig. 4b). We modelled a single phosphorylated site 3a (Ser641) (Fig. 2c), which was the first C-terminal phosphorylation site in the sequence (Fig. 2a). The density of this region was symmetric in both the *C1* and *D2* symmetry maps (Extended Data Fig. 4c) and probably represents an average of different conformations of the C termini. However, aided by both unfiltered and LAFTER denoised maps (Extended Data Fig. 4c), we modelled C-terminal residues from Pro637 to Val642 for one subunit and from Pro637 to Ser641 for the other across the dimeric interface (Fig. 2c). This clearly showed that Arg588 and Arg591 from both subunits could sense the phosphorylation of a single 3a site at any time (Fig. 2c). This implied that the other C terminus from the dimeric interface was excluded by steric occlusion, and both C termini appeared to traverse away from the enzyme core, as evidenced by the map density (Extended Data Fig. 4d) and fuzzy protrusions from this region in the 2D classes (Extended Data Fig. 1e). Overall, our model suggests that the nonsymmetric interactions of a single phosphorylated site (3a) at the dimeric (C–D and A–B) interfaces, combined with intersubunit interactions of phosphorylated sites 2 and 2a across the interface, stabilize GYS1 in the inhibited state.

### Allosteric activation by Glc6P

**Fig. 3:**
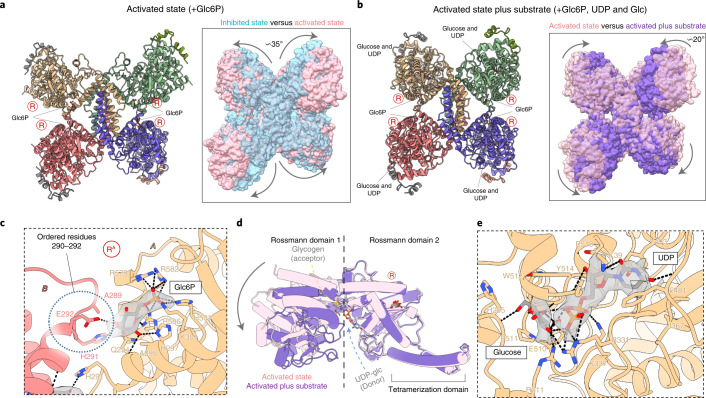
Activated structures of the phosphorylated R-state GYS1–GYG1^ΔCD^ complex with and without substrate. **a**, Structure of the Glc6P-bound activated (R) state determined from a 3.7 Å map. Inset shows the global conformational changes resulting from Glc6P activation in comparison with the inhibited (T) state. **b**, Structure of the R state bound to Glc6P, UDP and glucose determined from a 3.0 Å map. Inset shows the global conformational changes resulting from substrate binding in the activated state. Arginine clusters-containing regulatory α24 helices are labelled ‘R’. **c**, *Cis* and *trans* interactions with the Glc6P activator in the R state determined from the higher-resolution substrate-bound map. Interactions with Glc6P in the lower-resolution map without substrate were the same. Cryo-EM density for Glc6P is shown. **d**, Conformational changes of Rossmann domain 1 in relation to Rossmann domain 2 due to UDP and glucose binding in the R state. **e**, Interactions with UDP and glucose in the R state. Cryo-EM densities for both ligands are shown.

**Fig. 4:**
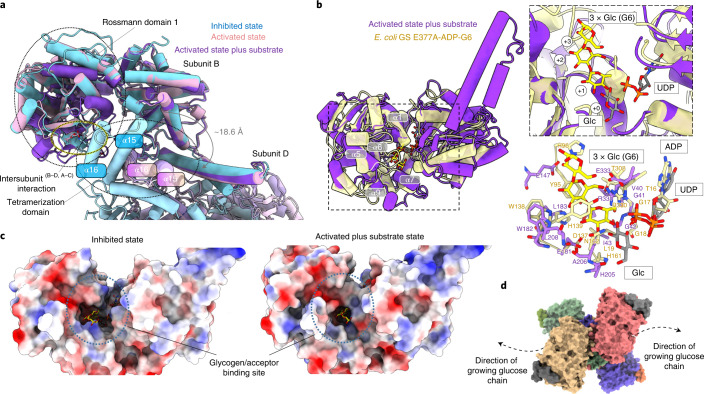
Structural comparison of the GYS1–GYG1^ΔCD^ inhibited and activated states with oligosaccharide-bound *E. coli* GS. **a**, Structural alignment of one subunit of the inhibited, activated and activated plus substrate-bound GYS1 structures. Tetramerization helices are highlighted to show relative movement between adjacent subunits within tetrameric GYS1. **b**, Structural alignment of the activated plus substrate-bound state against *E. coli* GS incubated with maltohexaose (G6) bound to three glucose moieties in the active site. The first inset shows the active site of the two structures. The second inset demonstrates conservation of key residues involved in glucan binding. **c**, Electrostatic surfaces of the inhibited and activated plus substrate-bound states. The predicted glycogen-binding site cleft is highlighted. **d**, Surface model of the activated state bound to UDP and glucose and the predicted direction of the growing glucose chain. *T*_m_, melting temperature. Source data

**Extended Data Fig. 5:**
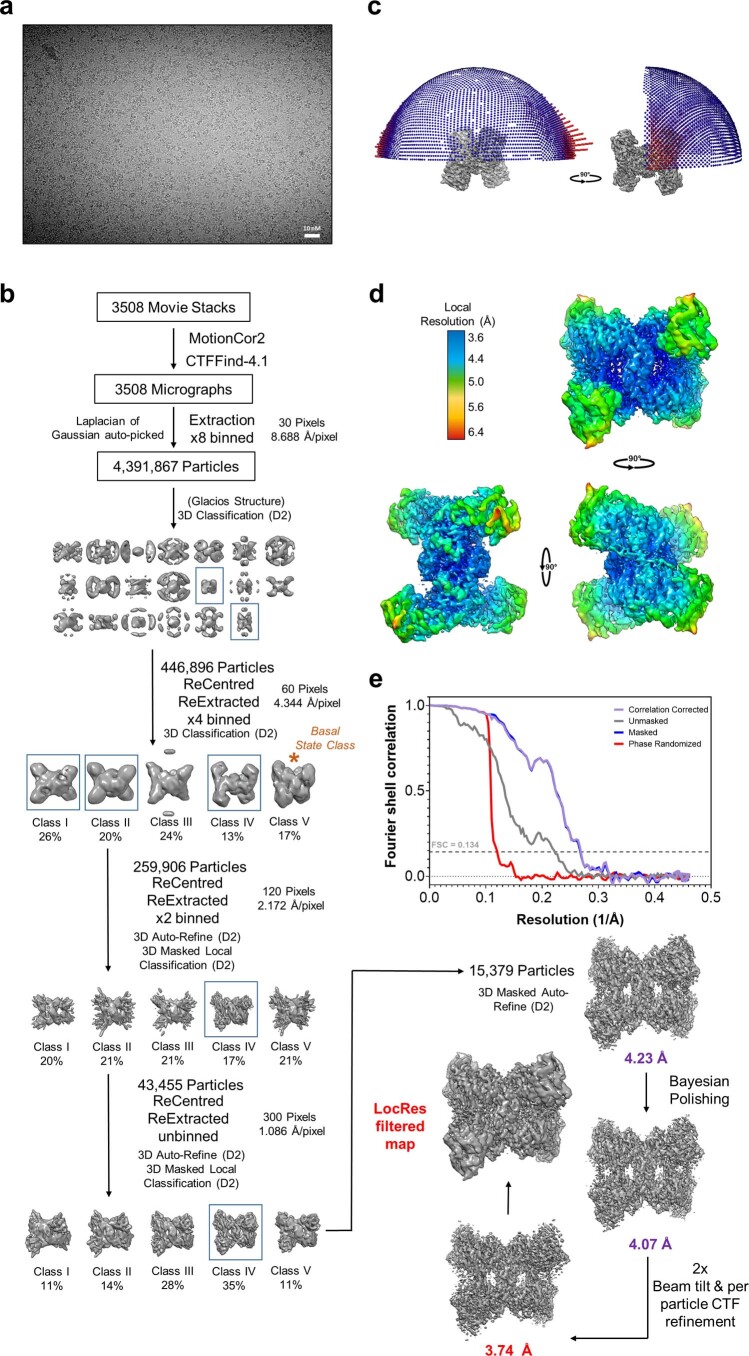
Image processing workflow of the GYS1:GYG1^ΔCD^ + Glc6P activated state. **a**, Representative K3 micrograph of the GYS1:GYG1^ΔCD^ + Glc6P activated state from 3508 micrographs collected. **b**, Processing flow chart of the GYS1:GYG1^ΔCD^ + Glc6P activated state. **c**, Angular distribution of the 3.74 Å GYS1:GYG1^ΔCD^ + Glc6P activated state map. **d**, Local resolution variation of the 3.74 Å GYS1:GYG1^ΔCD^ + Glc6P activated state map. **e**, FSC curve of the 3.74 Å GYS1:GYG1^ΔCD^ + Glc6P activated state map.

**Extended Data Fig. 6:**
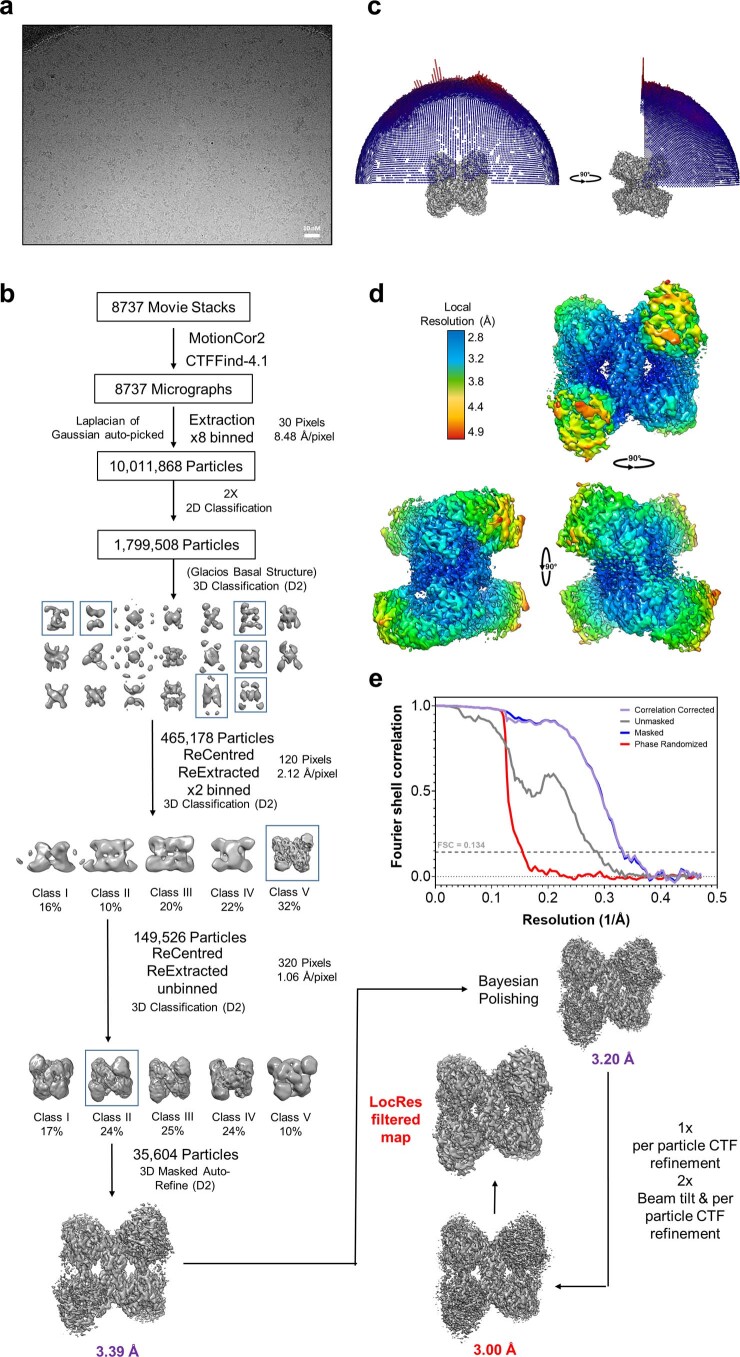
Image processing workflow of the GYS1:GYG1^ΔCD^ + Glc6P+UDP-glc activated state. **a**, Representative K3 micrograph of the GYS1:GYG1^ΔCD^ + Glc6P+UDP-glc activated state from 8737 micrographs collected. **b**, Processing flow chart of the GYS1:GYG1^ΔCD^ + Glc6P+UDP-glc activated state. **c**, Angular distribution of the 3.00 Å GYS1:GYG1^ΔCD^ + Glc6P+UDP-glc activated state map. **d**, Local resolution variation of the 3.00 Å GYS1:GYG1^ΔCD^ + Glc6P+UDP-glc activated state map. **e**, FSC curve of the 3.00 Å GYS1:GYG1^ΔCD^ + Glc6P+UDP-glc activated state map.

To reveal GYS1 in the R state, we determined a structure at 3.7 Å resolution in the presence of the allosteric activator Glc6P (Fig. 3a, Table 1, and Extended Data Fig. 5) and a structure of 3.0 Å resolution in the presence of both Glc6P and the sugar donor UDP-glc (Fig. 3b, Table 1, and Extended Data Fig. 6). Glc6P induced large global structural changes compared with the T state, resulting in an outward rotation of ~35° of each GYS1 subunit along the tetramer axis (Fig. 3a). This removed intersubunit contacts at the minor interfaces (B–D and A–C) between the N-terminal Rossmann domain 1 of one subunit and the tetramerization domain of the neighbouring subunit (Extended Data Fig. 3c), freeing access to the active site between the Rossmann domains. When aligning one GYS1 subunit each from the T and R states, the tetramerization domain of the neighbouring subunit (minor B–D and A–C interfaces) moved away by ~18.6 Å with respect to Rossmann domain 1 (Figs. 3a and 4a). The increased flexibility of the N-terminal Rossmann domain was evident from the EM map, as this region was of much lower resolution (~5.0 Å) than the core of the enzyme (~3.6 Å, Extended Data Fig. 5d).

Glc6P bound identically to both R-state structures, so we describe its binding mode based on the higher-resolution structure bound to Glc6P and UDP-glc (Fig. 3c). Arg579, Arg582 and Arg586 from the regulatory α24 helix, along with Lys301 and His501, were found to interact with the Glc6P phosphate moiety. The glucose moiety contacted His287, Gln294 and Arg586 from its own subunit (that is, in *cis*), along with the now-ordered residues His291 and Glu292 at the end of helix α13 from the neighbouring subunit across the dimeric interface (that is, in *trans*). The Glc6P binding mode and the disordered-to-ordered transition of residues 290–292 are conserved in the Glc6P-bound yeast Gsy2p structure^16^. Ordering of this region is essential for the transition from the basal or inhibited state to the activated state (next section).

The R state bound to UDP-glc adopted a similar conformation to that of the R state without UDP-glc (r.m.s.d. 0.71 Å), except for a rotation of ~20° of Rossmann domain 1 relative to Rossmann domain 2, which closed the active site cleft (Fig. 3b,d). We observed density at the sugar donor site, which fit better as individual UDP and glucose moieties, suggesting that UDP-glc was hydrolysed (Fig. 3e). This was similar to an activated structure of yeast Gsy2p incubated with UDP-glc, in which one subunit had UDP and glucose bound^17^. In our structure, the uridine moiety of UDP was sandwiched between Ile367, Phe481 and Tyr493, also forming a hydrogen bond with Lys19 (Fig. 3e). The Gly41 backbone and Glu518 side chain interacted with the ribose moiety, whereas Arg331 and Lys337 dispersed the charge of the diphosphate moiety. The hydrolysed glucose molecule formed multiple hydrogen bonds with the side chains of Arg211, Arg311, Glu510 and Tyr514, along with the backbones of His205, Trp512 and Gly513. In addition, Ala206 and Pro511 formed hydrophobic interactions with the sugar (Fig. 3e).

This UDP-glc-bound R state is predicted to be the catalytic competent state, which is poised for binding to the glucose chain substrate^29,35^. The map features of the N-terminal Rossmann domain 1 were blurred (Extended Data Fig. 6d), suggesting increased flexibility. To gain further insight into substrate binding and catalysis, we aligned one subunit of each of our states with the structure of the *E. coli* glycogen synthase (GS) incubated with maltohexaose, resulting in three glucose moieties bound to the active site (PDB 3CX4)^35^. The *E. coli* GS was in a closed conformation and aligned with r.m.s.d. values of 1.09 Å and 1.19 Å against our GYS1 inhibited and activated states, respectively (Fig. 4b). The GS glucose moieties occupied the +1 to +3 sites, whereas the hydrolysed glucose in our EM map occupied the +0 site (Fig. 4b). This predicted binding pocket of the glucan had conserved residues between *E. coli* GS and human GYS1 (Fig. 4b), suggesting that the initial growing glucose chain is threaded into and then out of the GYS1 active site through a cleft formed by helices α1, α5, α6, α7 and α9 of Rossmann domain 1 (Fig. 4b,d). This pocket was not closed in the T state and may explain the large increase in affinity for UDP-glc^36^ and glycogen when GYS1 is in the R state^37^ (Fig. 4c).

### Phosphorylation attenuates activation by Glc6P

**Fig. 5:**
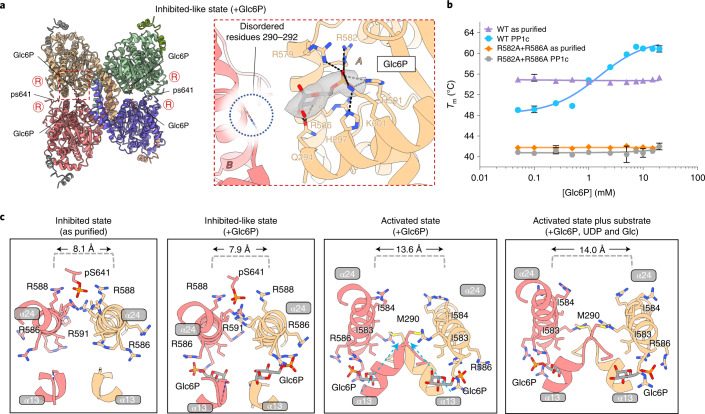
Phosphorylation hinders transition into the activated (R) state as shown by the phosphorylated inhibited (T) state bound to Glc6P. **a**, Overall model of the phosphorylated T state bound to Glc6P and the interactions with this activator. Inset shows cryo-EM density for Glc6P. Arginine clusters-containing regulatory α24 helices are labelled ‘R’. **b**, Thermal shift assay of as-purified (phosphorylated) versus PP1c-treated (dephosphorylated) GYS1–GYG1^ΔCD^ (labelled WT) and GYS1^p.R582A+p.R586A^–GYG1^ΔCD^ (labelled R582A + R586A) complexes in the presence of increasing concentrations of Glc6P. Median melting temperatures and standard deviations are shown (*n* = 4 technical repeats). **c**, Regulatory helix interactions and conformational changes as seen in our cryo-EM structures. Key residues are labelled. Distances between the regulatory α24 helices were determined as the distances between the Cα atoms of the Asn587 residues.

**Extended Data Fig. 7:**
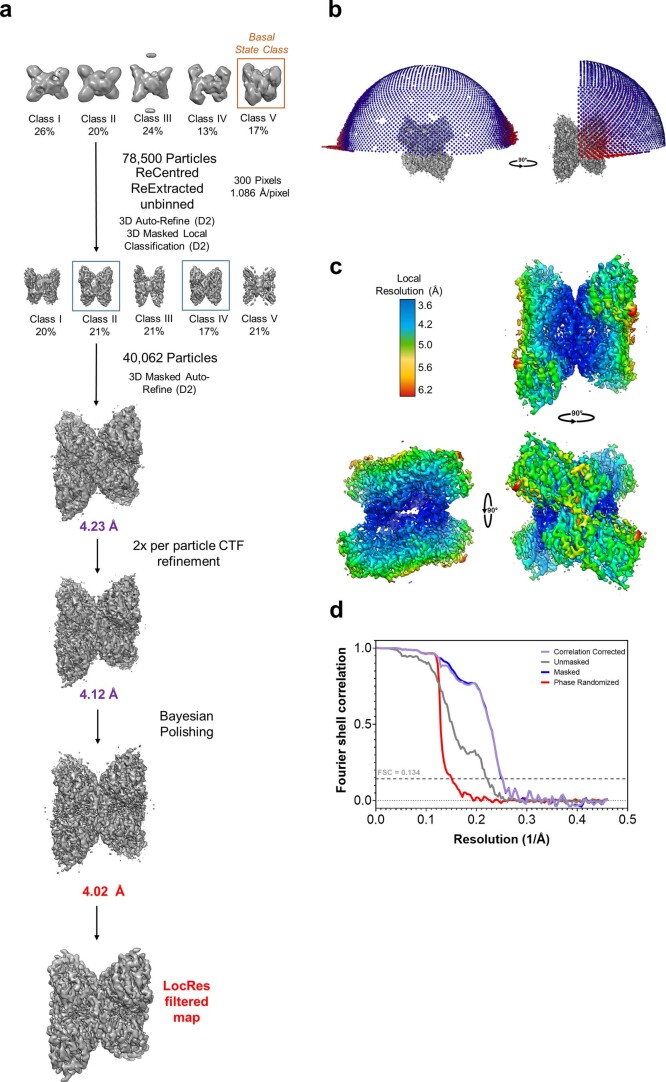
Image processing workflow of the GYS1:GYG1^ΔCD^ + Glc6P inhibited-like state. **a**, Processing flow chart of the GYS1:GYG1^ΔCD^ + Glc6P inhibited-like state. **b**, Angular distribution of the 4.02 Å GYS1:GYG1^ΔCD^ + Glc6P inhibited-like state map. **c**, Local resolution variation of the 4.02 Å GYS1:GYG1^ΔCD^ + Glc6P inhibited-like state map. **d**, FSC curve of the 4.02 Å GYS1:GYG1^ΔCD^ + Glc6P inhibited-like state map.

While processing the GYS1–GYG1^ΔCD^ + Glc6P data set, we observed that one three-dimensional (3D) class appeared similar to the inhibited (T) state and was refined to 4.0 Å resolution (Table 1 and Extended Data Fig. 7). Similar to our T state map, where phosphorylated Ser641 of the C terminus interacted with the arginine clusters, density for Glc6P in the allosteric site was apparent for this structure (Fig. 5a). Unlike the activated (R) state, Glc6P in this structure did not interact with subunits across the dimeric interface, because residues 290–292 remained disordered. In this ‘inhibited-like’ state, all interactions involved the phosphate group and were identical to the activated states except for Arg586, which was not in a productive conformation to interact with both glucose and phosphate moieties of Glc6P (Fig. 5c).

**Extended Data Fig. 8:**
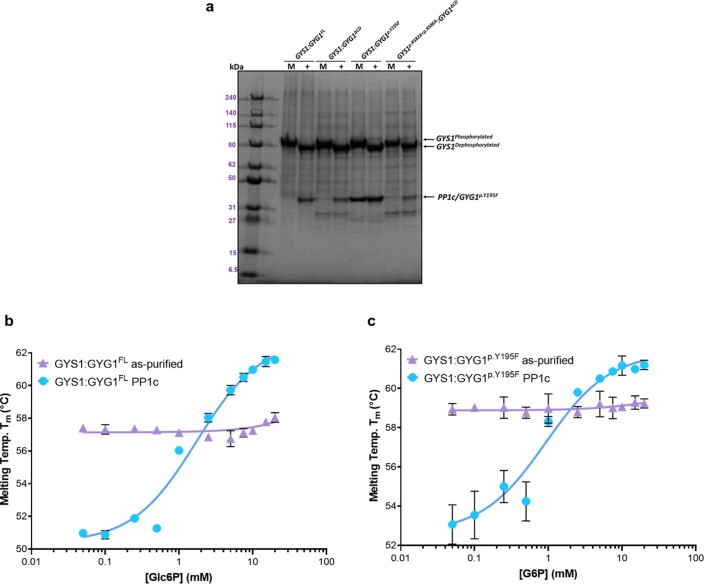
Thermal shift assay of phosphorylated (as purified) versus dephosphorylated (PP1 treated) GYS1:GYG1 complexes in the presence of increasing concentrations of Glc6P. **a**, Gel shift of GYS1:GYG complexes mock (M) or treated with PP1c (+) for 2 hours at room temperature. 5 µg of each complex was loaded and ran on SDS-PAGE. A decrease in the molecular weight of GYS1 after PP1 treatment is apparent. **b**, Thermal shift assay of GYS1:GYG1^FL^ against Glc6P. **c**, Thermal shift assay of GYS1:GYG1^p.Y195F^ against Glc6P. Median melting temperatures and standard deviations are shown (*n* = 4). Source data

This ‘inhibited-like’ state potentially exists in dynamic equilibrium with the activated state. Glc6P binding is well known to overcome the inhibitory effects of phosphorylation; however, reported *K*_a_ values of Glc6P for phosphorylated enzymes vary between 0.33 and 1.8 mM for insect-cell-expressed human GYS1 (refs. ^32,33^) and between 0.8 and 1.9 mM for rabbit GYS1 (ref. ^38^). Dephosphorylation appreciably reduces the amount of Glc6P to half-maximally activate the enzyme (A_50_) within a range of ~3-, ~10- or ~100-fold^39^. These diverse values are likely to reflect the phosphorylation heterogeneity of each sample and suggest an interplay between phosphorylation and Glc6P activation. Using the thermal shift assay, we titrated Glc6P against our three complexes (GYS1–GYG1^FL^, GYS1–GYG1^ΔCD^ and GYS1–GYG1^p.Y195F^), each in the as-purified (phosphorylated) and PP1c-treated (shown to partially dephosphorylate the protein, particularly at key sites^19^) forms (Extended Data Fig. 1f). For all three complexes, dephosphorylation considerably reduced thermostability by ~6 °C (Fig. 5b and Extended Data Fig. 8). This suggests that the phosphorylated T state is more stable than the dephosphorylated basal (I) state, probably owing to the loss of stabilizing interactions of phosphorylated residues with the arginine clusters. Notably, for all three constructs, Glc6P had no or little stabilizing effect towards phosphorylated complexes, whereas each dephosphorylated complex was readily stabilized by Glc6P, with a maximal increase in thermostability of ~8–12 °C (Fig. 5b and Extended Data Fig. 8). The apparent AC_50_ values (the concentration of ligand to reach half-maximal melting temperature) for each dephosphorylated construct were 1.7 ± 0.2 mM (GYS1–GYG1^FL^), 1.5 ± 0.2 mM (GYS1–GYG1^ΔCD^) and 0.9 ± 0.2 mM (GYS1–GYG1^p.Y195F^). These values are lower than the reported *K*_a_ values for dephosphorylated GYS1, probably owing to differences in the remaining phosphorylation of the samples and/or pleiotropic effects from substrates^39^. Furthermore, a GYS1^p.R582A+p.R586A^–GYG1^ΔCD^ complex, in which two arginines that interact with the Glc6P phosphate moiety were substituted, showed no stabilizing effect when treated with PP1c, confirming the critical role of these residues in binding Glc6P (Fig. 5b).

Next, we compared the orientations of regulatory α24 helices among our four structures (Fig. 5c). The ordering of residues 290–292 at the end of helix α13 (which interact with Glc6P) appeared to be the driver of conformational change resulting in enzyme activation. The ordering of these residues was associated with movement of helix α13 towards the regulatory α24 helix across the dimeric interface, positioning the hydrophobic Met290 (from α13) to interact with Ile583 and Ile584 (from α24). This drives apart the regulatory helices across the dimeric interface, distancing them from 8.1 Å to 13.6 Å and abolishing the interactions of Arg588 and Arg591 from both subunits with the single phosphorylated Ser641. This allows for greater flexibility between each subunit, as the distance increases further to 14.0 Å when the sugar donor is present (Fig. 5c).

**Extended Data Fig. 9:**
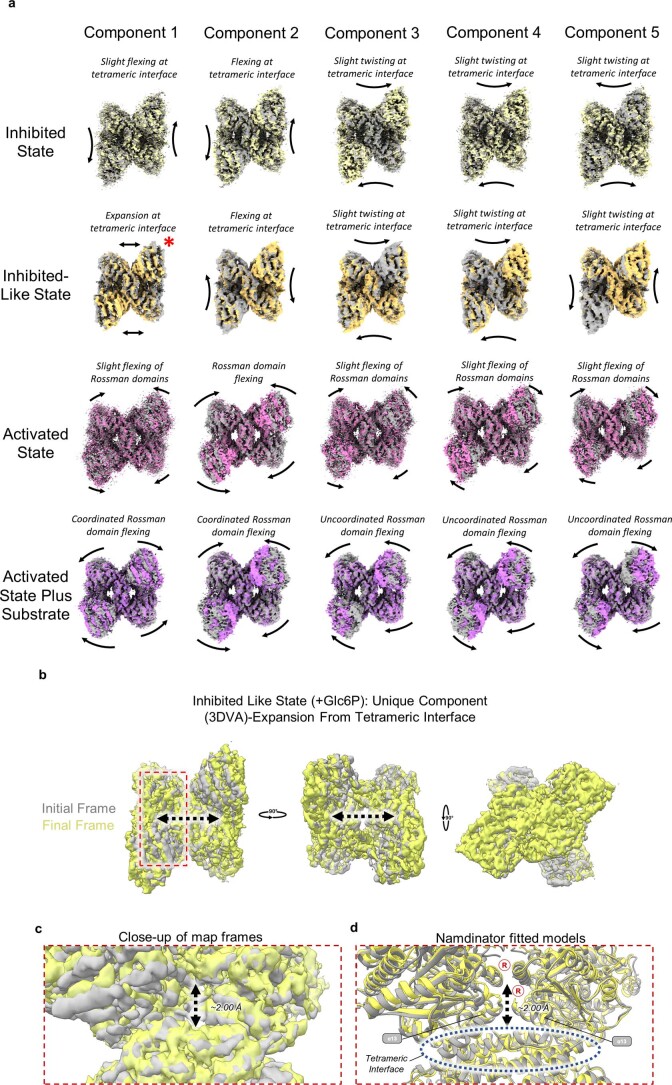
3D variability analysis of the four different states of GYS1 and the unique component of the inhibited like Glc6P bound state. **a**, 3D variability analysis components of all four states of GYS1 reported in this study. Initial and final frames are shown. The unique component of the inhibited like-state is highlighted by a red asterisk. Most movements are either slight flexing at the tetrameric interface or flexing of the N-terminal Rossman domains. **b**, Alignment of initial and final frames showing a global expansion from the central helical tetrameric interface. **c**, Close-up of the frames around the allosteric/G6P binding density. **d**, Namdinator fitted models into the initial and final frames showing a clear movement of the alpha-helices 13 from both subunits towards the regulatory helices.

In addition, 3D variability analysis of the four structures revealed that the R states are far more flexible than the T states (Extended Data Fig. 9a and Supplementary Videos 1–5). In both R states, the Rossmann domain 1 flexed onto Rossmann domain 2. This movement was more pronounced when substrate was bound to the active site. No such Rossmann domain closure was apparent in either T state. However, 3D variability analysis of the Glc6P-bound inhibited-like state showed a unique movement not observed in the inhibited state without Glc6P. This appeared as a 2.0 Å expansion of the complex from the tetrameric interface (Supplementary Video 1, Extended Data Fig. 9b); by flexibly fitting our inhibited-state model, we observed that helix α13 moved towards the regulatory helices (Extended Data Fig. 9c,d). This suggests that the inhibited-like state is primed to change to the activated state, by either changes in dynamic equilibrium, binding of substrate and/or dephosphorylation by PP1. These findings, coupled with our thermal shift results, suggest that the conformational change to the activated state is attenuated by the phosphorylation of site 3a and possibly 2 and 2a.

### Associated glycogen of the GYS1–GYG1 complex recruits PTG

**Fig. 6:**
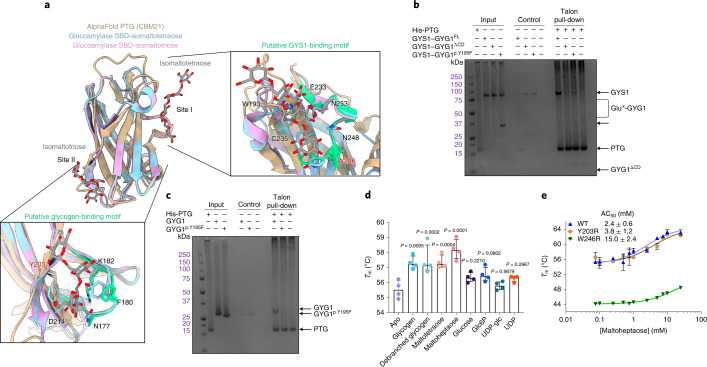
CBM21 domain of PTG binds to the GYS1–GYG1 complex via the associated glucose chain. **a**, Structural alignment of the AlphaFold predicted structure of the PTG(CBM21) domain against the starch-binding domain (SBD) from *R. oryzae* glucoamylase bound to maltotetraose and maltotriose at site I and site II, respectively. Panels show how site I and site II align with the putative GYS-binding motif and putative glycogen-binding motif. Both motifs are coloured green. Y203 and W246 labels are highlighted red. **b**, PTG(CBM21) was incubated with GYS1–GYG1^FL^, GYS1–GYG1^pY195F^ or GYS1–GYG1^ΔCD^. The ability of PTG to bind GYS1–GYG1 complexes was assessed by affinity pull-down, followed by SDS–PAGE (*n* = 4 technical repeats). **c**, PTG(CBM21) was incubated with GYG1 or GYG1^p.Y195F^ catalytic domain constructs, passed onto affinity resin and analysed by SDS–PAGE (*n* = 4 technical repeats). The GYG1 catalytic domain exists as a mixture of glucosylated states and runs at a higher apparent molecular weight in SDS–PAGE than GYG1^p.Y195F^, which is nonglucosylated. **d**, Thermal shift analysis of PTG(CBM21) in the presence of various sugars and ligands (*n* = 4 technical repeats). *P* values between the apo and plus sugar samples were determined by two-tailed unpaired *t*-test. **e**, Thermal shift analysis of PTG(CBM21) wild type (labelled WT), PTG(CBM21)^p.Y203R^ variant (labelled Y203R) and PTG(CBM21)^p.W246R^ variant (labelled W246R) in the presence of increasing concentrations of maltoheptaose. Median melting temperatures (*T*_m_) and standard deviations are shown (*n* = 3 technical repeats). Source data

PP1 dephosphorylates GYS1 in vivo with assistance from a glycogen-targeting regulatory protein, such as PTG, which has been suggested to directly interact with GYS1 (ref. ^19^). Attempts to express full-length human PTG were unsuccessful; we instead obtained soluble protein with a construct encompassing Leu134–Val259 that contained the carbohydrate-binding module 21 (CBM21) domain (residues 149–257). Using the AlphaFold^40^ predicted model of this domain, we overlaid two crystal structures of the starch-binding domain from *Rhizopus oryzae* glucoamylase bound to maltotriose and maltotetraose at two different sites (starch-binding sites I and II)^41^. The *R. oryzae* sites I and II aligned well with the PTG(CBM21) regions harboring the putative glycogen-binding (VKNVSFEKKV, residues 175–184) and GYS-binding (WDNNDGQNYRI, residues 246–256) sequences, respectively (Fig. 6a). In addition, sequence alignment of all known glycogen-targeting PPP1R3 regulatory subunits against the starch-binding domain of *R. oryzae* glucoamylase showed that both VxNxxFEKxV and WxNxGxNYx(I/L) motifs are highly conserved across all the CBM21 domains, suggesting that both motifs in PTG(CBM21) are involved in glycogen binding (Supplementary Fig. 2) and that PTG(CBM21) does not interact physically with GYS1.

We further used affinity pull-down to evaluate the binding of PTG(CBM21) to GYS1–GYG1 complexes (Fig. 6). His-tagged PTG(CBM21) pulled down only GYS1–GYG1^FL^, where GYG1 is attached with a glucose chain (glucosylated); it did not pull down the GYS1–GYG1^ΔCD^ or GYS1–GYG1^p.Y195F^ complexes, where GYG1 is not glucosylated (Fig. 6a and Supplementary Fig. 3a). This was consistent with analysis by blue-native polyacrylamide gel electrophoresis (PAGE) (Supplementary Fig. 3c), suggesting that PTG(CBM21) is recruited to GYS1 by the GYG1-associated glycogen. To confirm a direct interaction between PTG and the GYG1 glucose chain, we repeated the PTG pull-down with the GYG1 catalytic domain alone from the wild type (glucosylated) and GYG1^p.Y195F^ (nonglucosylated), without GYS1. His-tagged PTG(CBM21) pulled down only the glucosylated GYG1 catalytic domain, not the nonglucosylated GYG1^p.Y195F^ (Fig. 6b and Supplementary Fig. 3b).

Next, the polysaccharide-binding ability of PTG(CBM21) was studied by thermal shift assay. Glycogen, debranched glycogen, maltotetraose and maltoheptaose increased the thermostability of PTG(CBM21) (Fig. 6d). To delineate PTG(CBM21) sequences that were involved in sugar binding, we substituted to arginine the residues Tyr203 and Trp246, representing a conserved residue within the equivalent site II and site I of *R. oryzae*, respectively (Fig. 6a). Whereas PTG(CBM21)^p.Y203R^ had a similar melting temperature to the wild type, PTG(CBM21)^p.W246R^ was approximately 10 °C less stable (Fig. 6e). Titrating maltoheptaose stabilized both wild-type PTG(CBM21) and PTG(CBM21)^p.Y203R^ similarly, with AC_50_ values of 2.4 ± 0.6 mM and 3.8 ± 1.2 mM, respectively. PTG(CBM21)^p.W246R^ had a severely reduced ability to bind maltoheptaose, with an apparent AC_50_ of 15.0 ± 2.4 mM (Fig. 6e), showing that site I has a substantial role in sugar binding. Overall, these results suggest that the GYG1-associated glycogen of the GYS1–GYG1 complex is the major binding site of PTG and that any direct GYS1-PTG interactions are potentially quite weak, outside the CBM21 domain or only form in the presence of PP1.

## Discussion

Our cryo-EM structures have unraveled the role of phosphorylated N and C termini as a molecular ‘straitjacket’, reducing the flexibility of the GYS1 tetramer and hindering Glc6P-mediated conformational change to the activated state. Specifically, phosphorylated site 3a (and potentially also sites 2 and 2a) is poised to interact with arginine clusters at the dimeric interface, confirming their importance relative to other sites^39^. Sites 2 and 2a could interact with Arg579 and Arg580 in *trans* (across the dimer interface). Unexpectedly, one single phosphorylation at site 3a interacts with Arg588 and Arg591 from both subunits at the dimeric interface (that is, both in *cis* and in *trans*). The essentiality of Arg579, Arg580, Arg588 and Arg591 for phosphorylation-dependent inhibition is supported by mutagenesis of equivalent residues in yeast Gsy2p^16^ and mouse GYS1 (refs. ^39,42,43^). This is underscored by reciprocal mutagenesis of sites 2 and 2a and 3a in rabbit GYS1 that ablated inhibition by phosphorylation^12,44^ and/or improved sensitivity toward Glc6P activation^45^. The relative contributions of site 2 and 2a and site 3 in inducing phosphorylation-dependent inhibition remain unclear, and translating biochemical findings from yeast, mouse and rabbit orthologues to understanding the human enzyme may also be hindered by the variation in the lengths and sequences of their N termini^16,38,39^.

The Glc6P binding site, involving Arg579, Arg582 and Arg586 of the arginine cluster, is highly conserved between yeast and human^16^. Particularly, the importance of Arg582 and Arg586 is confirmed by their substitution in rabbit and yeast GYS, which abolished Glc6P activation^16,39,42,43^, consistent with our findings for human GYS1 (Fig. 5b). The Glc6P-induced conformational change is also conserved in yeast Gsy2p^16^, and our four human structures clarify that the ordering of residues Met290–Glu292 to interact with Glc6P in *trans* across the dimer interface drives the conformational change. This positions Met290 between the two regulatory α24 helices at the dimer interface, driving them apart with steric hinderance against Ile583 and Ile584 of the *trans* subunit. Therefore, Glc6P activation replaces the ionic interaction of phosphorylation with a hydrophobic interaction, allowing for greater flexibility between subunits and between the Rossmann domains from a single subunit, thereby increasing active site access. The equivalent residues of Met290, Ile583 and Ile584 in yeast (Phe299, Ile584 and Asn585) and *C. elegans* (Leu308, Ile604 and Ile605) suggest a shared mechanism for allosteric activation of glycogen synthase as a homotetramer.

Dephosphorylation by PP1 also relieves inhibition of GYS1 by removing the phosphorylation at sites 2 and 2a and 3a, thus releasing the ‘straitjacket’ effects of the N and C termini^32,33,39^. PP1 is recruited to glycogen by seven regulatory subunits^46^, among which PTG is ubiquitously expressed^47^ and considered a therapeutic target for GSDs^22^. All glycogen-recruiting regulatory subunits share a PP1-binding motif and a CBM21 domain^21^. The latter contains two putative binding sites^20^, namely site II, which corresponds to a glycogen-binding motif^19–21^, and site I, which has been suggested to be a GYS-binding motif based on work on the CBM21 domain of muscle-specific PPP1R3A (with 65.7% sequence similarity to PTG)^19,48^ (Supplementary Fig. 2). Our pull-down experiments show that PTG(CBM21) does not interact directly with GYS1, in contrast to a recent study involving PPP1R3A and the full-length GYS1–GYG1 complex that did not account for GYG1 self-glucosylation^32^. Instead, our mutagenesis results mirror previous findings on *R. oryzae* glucoamylase, where mutating the equivalent residue (Tyr94, corresponding to Trp246 in PTG) in site I severely reduced the binding affinity for carbohydrate^49^. These findings suggest that PTG (and possibly other glycogen-targeting PP1 regulatory subunits) recruits PP1 to GYS1 via the GYG1-attached glucose chain. With multiple surface sites in addition to the active site of GYS1 for glycogen contacts^15^, the PTG-glycogen interaction therefore provides for GYS1 processivity, by facilitating PP1 recruitment to flexibly dephosphorylate^50^ the many sites on the GYS1 N and C termini. However, a GYS1-binding site could be formed in full-length PTG or in complex with PP1; therefore, further investigation is needed.

**Fig. 7:**
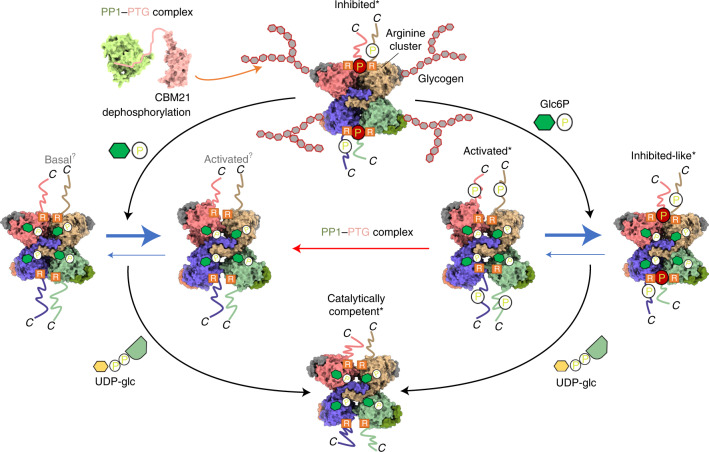
Proposed model of phosphorylation and Glc6P regulation of GYS1 activity. Only the C termini and 3a phosphorylation site are shown for simplicity. In addition, the associated glycogen is only shown for the inhibited state, although it is present in all other states. Asterisk indicates structures that have been experimentally determined. Question mark indicates theoretical structures. Our model based on the structural data proposes that the inhibited (T) state is catalytically inactive because the phosphorylated N and C termini bind to a subunit interface. This locking interaction reduces GYS1 flexibility and prevents active site closure by the two Rossmann domains. Glc6P binding to the allosteric site overcomes these inhibitory effects to promote a conformational change to the activated (R) state. However, the R state is in a dynamic equilibrium with an inhibited-like state, owing to competition between the locking interactions of phosphorylated termini at the subunit interface and the conformational change due to Glc6P binding. The inhibition of phosphorylation can also be relieved by the concerted actions of the PP1–PTG complex that binds to the associated glycogen and dephosphorylates the GYS1 N and C termini, resulting in the basal (I) state. This intermediate state is more susceptible to the allosteric effects of Glc6P binding, shifting the dynamic equilibrium more toward the activated state. In the activated state, binding of the substrate UDP-glc promotes the closure of the cleft between the two Rossmann domains, resulting in a catalytically competent state for extending the associated glycogen chain.

Our structural snapshots reveal a model of GYS1 regulation by both Glc6P and phosphorylation, explaining how their interplay alters the equilibrium of the various GYS1 states, further elaborating the lock-and-key hypothesis of these two effectors (Fig. 7)^16,39^. This dynamic equilibrium is likely to fine-tune glycogen formation, responding to upstream messengers such as insulin^14^. Furthermore, our structures provide opportunities for rational inhibitor design in the development of new GSD therapies. GYS1 as a target is validated by proof-of-concept *GYS1* knockout in cell and animal models^23,50^, and a safety profile is underscored by healthy individuals with reduced GYS1 enzyme activity^51,52^. Preventing dephosphorylation by targeting PTG or the Glc6P allosteric site are appealing starting points for inhibitor design. Indeed, ATP has been suggested to be a competitive inhibitor of Glc6P and may trap GYS1 in an inhibited state^39^. Overall, our structural work elucidates the results of decades of studies on the arginine clusters, key phosphorylation sites and conformational flexibility of GYS1 (Table 1).

## Methods

### Cloning, expression and purification of GYS1–GYG1 complexes

DNA sequences of the full-length human *GYS1* (IMAGE: 3143019) and *GYG1* (IMAGE: 3504538; isoform GN-1L with UniProt ID P46976-1) genes were amplified from a complementary DNA clone and subcloned into the FastBac-Dual vector (Life Technologies) with an N-terminal His_6_-tag and a TEV protease cleavage site on GYS1. The GYG1^p.Y195F^-expressing mutant was generated from this plasmid using the QuickChange mutagenesis kit (Stratagene). Codon-optimized genes encoding GYS1 and aa 264–350 or 294–350 GYG1 (GYG1^ΔCD^) (with a stop codon) interspersed with a SV40 terminator and a polyhedrin promotor were artificially synthesized (Twist Biosciences). Codon-optimized sequences for either a N-terminal TEV-cleavable MBP-His_6_, His_6_-GST or His_6_-GFP tag were appended to the *GYG1* gene to allow purification. The resulting bicistronic fragment was then inserted into pFB-CT10HF-LIC for insect cell expression. In-Fusion HD (Takara) mutagenesis was used to introduce specific mutations in the coding region of *GYS1*. All GYS1–GYG1 complexes were expressed in Sf9 cells grown in Sf-900 III SFM (Life Technologies). Cell pellets were harvested and homogenized in lysis buffer (50 mM sodium phosphate pH 7.5, 500 mM NaCl, 5% glycerol, 0.5 mM Tris(2-carboxyethyl)phosphine hydrochloride (TCEP), 10 mM imidazole), and insoluble material was removed by centrifugation. The GYS1–GYG1 complexes were purified by affinity (Ni-Sepharose; GE Healthcare) and size-exclusion (Superose 6; GE Healthcare) chromatography. Protein was treated with His-tagged TEV protease overnight at 4 °C and then passed over Ni-Sepharose resin to remove the TEV protease and uncleaved protein. Purified complexes were concentrated to 10–20 mg ml^−1^ and stored in storage buffer (25 mM HEPES pH 7.5, 500 mM NaCl, 5% glycerol, 0.5 mM TCEP) at −80 °C.

### Cryo-EM sample preparation and data acquisition

GYS1–GYG1^ΔCD^ was diluted to 0.75 mg ml^−1^ in 25 mM HEPES, pH 7.5, 200 mM NaCl, 2.0 mM TCEP and 0.05% (v/v) Tween-20 for the as-purified, inhibited state. For the activated states, GYS1–GYG1^ΔCD^ was diluted to 0.75 or 0.5 mg ml^−1^ in 25 mM HEPES, pH 7.5, 200 mM NaCl, 2.0 mM TCEP, 0.05% (v/v) Tween-20, 5 mM Glc6P and 5 mM UDP-Glc when appropriate. Grids were prepared using a FEI Vitrobot Mark III at 4 °C and 100% humidity. Sample (3 µl) was applied to a plasma-treated gold coated R 1.2/1.3 300 mesh holey carbon grid (Quantifoil), with a blot force of 0, a blot time of 3 s and a wait time of 10 s.

Movies of GYS1–GYG1^ΔCD^ as purified and in the presence of Glc6P were collected during the same session at the Midlands Regional Cryo-EM Facility on a FEI Titan Krios equipped with a K3 (Gatan) direct electron detector operating in super-resolution mode. Images were obtained at 300 kV with a magnification of 81,000×, corresponding to a physical pixel size of 1.086 Å (super-resolution pixel size of 0.543 Å). Forty-five frames over 5 s were recorded with a defocus range of –0.8 µm to –2.3 µm with a total dose of 1.22 e^−^ A^−2^ per frame. Movies of GYS1 + GYG1^ΔCD^ in the presence of Glc6P and UDP-Glc were collected at eBIC (Diamond Light Source) on a FEI Titan Krios equipped with a K3 (Gatan) direct electron detector operating in super-resolution mode. Images were obtained at 300 kV with a magnification of 81,000×, corresponding to a physical pixel size of 1.06 Å (super-resolution pixel size of 0.53 Å). Fifty frames over 3.4 s were recorded with a defocus range of –0.8 µm to –2.3 µm with a total dose of 1.00 e^–^ A^–2^ per frame.

All data sets were corrected for beam-induced motion with MotionCor2 (ref. ^53^), and the contrast transfer function (CTF) was estimated using CTFFIND-4.1 (ref. ^54^). Particles were autopicked using Relion 3.1.1 (ref. ^55^). The Laplacian of Gaussian function and all further processing were done in Relion 3.1.1. For more detailed information on the processing workflow for all data sets, please see Extended Data Figs. 2 and 5–7. All final maps were automatically sharpened in Relion 3.1.1 and, for all but the inhibited state, locally filtered by resolution using LocRes. LAFTER^56^ maps were produced in aid of model building. Relion-extracted particles and maps were imported into CryoSPARC v. 3.1.0 to use for 3D variability analysis^57^ with five components. Components were visualized by a 3DVA simple display with 20 frames each using UCSF Chimera.

### Model fitting, refinement and validation

Initial models of human GYS1 and GYG1 were built using the SWISS-MODEL server^58^ with structures of the *C. elegans* gsy-1–gyg-1^ΔCD^ and the activated Glc6P-bound state of yeast Gsy2p (PDB 4QLB and 3NB0, respectively) as templates. GYS1 models were docked into maps using Molrep^59^, and GYG1 was manually docked using UCSF Chimera. For the GYS1–GYG1^ΔCD^ + Glc6P+UDP-glc activated state map, Namdinator^60^ was used to flexibly fit the refined GYS1–GYG1^ΔCD^ + Glc6P activated model. Further model building and manual refinement were performed in COOT^61^, followed by iterative cycles of real-space refinement in Phenix^62^. Final models were validated using MolProbity^63^. Figures were created in UCSF Chimera and Chimera X^64^.

### Dephosphorylation of GYS1–GYG1 complexes

GYS1–GYG1 complexes at 5.0 mg ml^−1^ were dephosphorylated with 0.5 mg ml^−1^ PP1c in 25 mM HEPES, pH 7.5, 200 mM NaCl, 2.0 mM TCEP and 2.0 mM MnCl_2_ at room temperature for 1 h. Reactions were halted by putting them into ice.

### UDP-Glo activity assay

The activity of GYS1–GYG1 complexes was measured using the UDP-Glo GT (Promega) according to the manufacturer’s instructions. To measure activity, 10 μl per well of each reaction containing 100 nM GYS1–GYG1, 1 mM UDP-glc, 0.5 mg ml^−1^ glycogen and 10 mM Glc6P in assay buffer (25 mM HEPES, pH 7.5, 200 mM NaCl, 0.5 mM TCEP) was dispensed into 384-well assay plates (Greiner). Following a 60-min incubation at room temperature, 10 μl of UDP-Glo Plus detection reagent was added (final assay volume: 20 μl per well) and, after a further 60 min of room-temperature incubation, luminescence was detected using a SpectraMax M3 (Molecular Devices).

### Cloning, expression and purification of GYG1 and PTG

A DNA fragment encoding human PTG (PPP1R3C) aa 134–259 (IMAGE clone: 4245774) was subcloned into the pNIC28-Bsa4 vector (GenBank accession no. EF198106) incorporating an N-terminal TEV-cleavable His_6_-tag. In-Fusion HD (Takara) mutagenesis was used to introduce specific mutations in the coding region of *PTG*. Both GYG1 (ref. ^65^) and PTG were cultured in autoinduction Terrific Broth (Formedium) at 37 °C and induced overnight at 18 °C. Cell pellets were harvested, homogenized in lysis buffer (50 mM sodium phosphate pH 7.5, 500 mM NaCl, 5% glycerol, 0.5 mM TCEP and 10 mM imidazole), and insoluble material was removed by centrifugation. The supernatant was purified by affinity (Ni-Sepharose; GE Healthcare) and size-exclusion (Superdex 75; GE Healthcare) chromatography. GYG1 was treated with His-tagged TEV protease overnight at 4 °C and then passed over Ni-Sepharose resin to remove the TEV protease and uncleaved protein. Purified protein was concentrated to 10–20 mg ml^−1^ and stored in storage buffer (25 mM HEPES pH 7.5, 500 mM NaCl, 5% glycerol, 0.5 mM TCEP) at −80 °C.

### Talon pull-down assay

His-PPP1R3C (1.0 mg ml^−1^) was preincubated with either GYS1–GYG1 complex (0.25 mg ml^−1^) or GYG1 (0.5 mg ml^−1^) for 30 min at 4 °C in a total volume of 100 μl. Next, 80 μl of a 50% slurry of Talon resin (Clontech) in binding buffer (25 mM HEPES, pH 7.5, 100 mM NaCl, 1 mM TCEP, 0.2% Tween-20) was added, followed by incubation for a further 30 min at 4 °C. The resin was washed with 2 ml binding buffer with 10 mM imidazole and eluted with 40 μl 4× sodium dodecyl sulfate (SDS)–PAGE sample buffer. Samples were run on SDS–PAGE and stained with Coomassie blue.

### Thermal shift assay

His-PPP1R3C or GYS1–GYG1 complex was diluted in thermal shift buffer (25 mM HEPES, pH 7.5, 200 mM NaCl, 2.0 mM TCEP) to 0.1 mg ml^−1^ with SYPRO-Orange (Invitrogen) diluted 1000× with ligand at 1 mM in a total volume of 20 μl. Protein with ligand was incubated for 5 min at room temperature in 96-well PCR plates before the addition of SYPRO-Orange. A Mx3005p real-time PCR machine (Stratagene) with excitation and emission filters of 492 and 610 nm, respectively, was used to measure temperature shifts. AC_50_ values (half-maximal effective ligand concentration) were determined by fitting the melting temperatures using GraphPad Prism (v. 9; GraphPad Software).

### Blue-Native PAGE

Blue-Native PAGE was carried out according to the manufacturer’s instructions (Life Technologies). His-PPP1R3C, GYS1–GYG1 complex and/or GYG1 were diluted in thermal shift buffer (25 mM HEPES, pH 7.5, 200 mM NaCl, 1.0 mM TCEP) preincubated for 5 min at room temperature. All blue-native PAGE experiments were performed three times independently.

### Reporting summary

Further information on research design is available in the Nature Research Reporting Summary linked to this article.

## Online content

Any methods, additional references, Nature Research reporting summaries, source data, extended data, supplementary information, acknowledgements, peer review information; details of author contributions and competing interests; and statements of data and code availability are available at 10.1038/s41594-022-00799-3.

## Supplementary information

Supplementary Information Supplementary Figs. 1–4, Supplementary Table 1, uncropped image for Supplementary Fig. 3a, uncropped gel image for Supplementary Fig. 3b, uncropped gel image for Supplementary Fig. 3c.

Reporting Summary

Peer Review File

Supplementary Video 1 3D variability analysis component 1 of all four states of GYS1.

Supplementary Video 2 3D variability analysis component 2 of all four states of GYS1.

Supplementary Video 3 3D variability analysis component 3 of all four states of GYS1.

Supplementary Video 4 3D variability analysis component 4 of all four states of GYS1.

Supplementary Video 5 3D variability analysis component 5 of all four states of GYS1.

## Data Availability

Structures and EM maps of GYS1–GYG1^ΔCD^ inhibited state (EMDB-13743, PDB 7Q0B), GYS1–GYG1^ΔCD^ + Glc6P inhibited-like state (EMDB-13751, PDB 7Q0S), GYS1–GYG1^ΔCD^ + Glc6P activated state (EMDB-13752, PDB 7Q12) and GYS1–GYG1^ΔCD^ + Glc6P+UDP-Glc activated state (EMDB-13753, PDB 7Q13) have been deposited in the Electron Microscopy Data Bank and Protein Data Bank. All main data supporting the findings of this study are available within the article, Extended Data and Supplementary Information. Source data are provided with this paper.

## Source data

Source Data Fig. 4 Determined melting temperatures for Fig. 4b.
Source Data Fig. 6 Uncropped gels for Fig. 6b,c.
Source Data Fig. 6 Determined melting temperatures for Fig. 6d,e.
Source Data Extended Data Fig. 1 Uncropped gels for Extended Data Fig. 1a–c.
Source Data Extended Data Fig. 1 Raw mass spectrometry data and UDP-Glo assay data for Extended Data Fig. 1f,g.
Source Data Extended Data Fig. 8 Uncropped gel for Extended Data Fig. 8a.
Source Data Extended Data Fig. 8 Determined melting temperatures for Extended Data Fig. 8b,c.
