# Supplementary material for: Molecular basis for the regulation of human glycogen synthase by phosphorylation and glucose-6-phosphate
Source: Nat Struct Mol Biol. 2022 Jul 14;29(7):628–38. doi: 10.1038/s41594-022-00799-3 (PMC9287172; doi:10.1038/s41594-022-00799-3)

nature portfolio

https://doi.org/10.1038/s41594-022-00799-3

Supplementary information

Molecular basis for the regulation of human glycogen synthase by phosphorylation and glucose-6-phosphate

In the format provided by the authors and unedited

Molecular basis for the regulation of human glycogen synthase by phosphorylation and glucose-6-phosphate

Thomas J. McCorvie[1,6], Paula M. Loria[2], Meihua Tu[3], Seungil Han[4], Leela Shrestha[1], D. Sean Froese[1,5], Igor M. Ferreira[1], Allison P. Berg[2 ✉], Wyatt W. Yue[1,6 ✉]

[1]Centre for Medicines Discovery, Nuffield Department of Clinical Medicine, University of Oxford, Oxford OX3 7DQ, UK
[2]Rare Disease Research Unit, Worldwide Research and Development, Pfizer Inc., 610 Main Street, Cambridge, MA 02139, USA
[3]Pfizer Inc. Medicine Design, Cambridge, Massachusetts 02139, USA
[4]Discovery Sciences, Worldwide Research and Development, Pfizer Inc., Eastern Point Road, Groton, CT 06340, USA
[5]Present address: Division of Metabolism and Children's Research Center, University Children's Hospital Zürich, University of Zürich, Switzerland
[6]Present address: Biosciences Institute, The Medical School, Newcastle University, Newcastle upon Tyne, NE2 4HH, UK

✉e-mail: allison.berg@pfizer.com; wyatt.yue@cmd.ox.ac.uk

Supplementary Figures

Supplementary Fig. 1: Sequence alignment of GYS proteins.

Supplementary Fig. 2: Sequence alignment of the N-terminus and CBM21 domain of each PP1 regulatory subunit and the starch binding domain of *R. oryzae* glucoamylase.

Supplementary Fig. 3: PTG pulldowns and BN-PAGE replicates.

Supplementary Fig. 4: Representative thermal shift unfolding curves of each GYS1:GYG1 complex and PTG(CBM21) mutant.

Supplementary Table 1: Sequences of oligonucleotides used for mutagenesis.

Uncropped image of Supplementary Fig. 3a

Uncropped image of Supplementary Fig. 3b

Uncropped image of Supplementary Fig. 3c

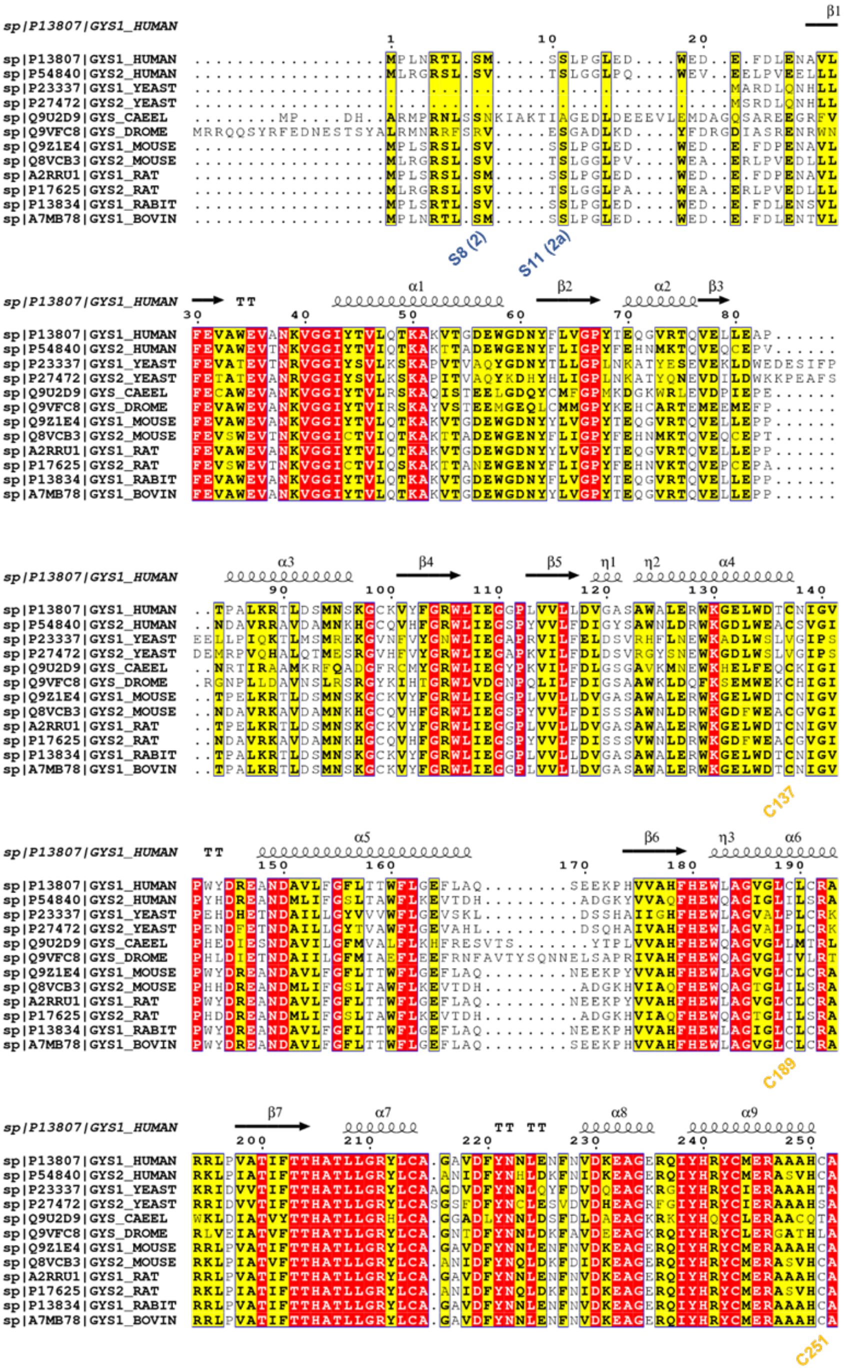
sp|P13807|GYS1_HUMAN
sp|P54840|GYS2_HUMAN
sp|P23337|GYS1_YEAST
sp|P27472|GYS2_YEAST
sp|Q9U2D9|GYS_CAEEL
sp|Q9VFC8|GYS_DROME
sp|Q9Z1E4|GYS1_MOUSE
sp|Q8VCB3|GYS2_MOUSE
sp|A2RRU1|GYS1_RAT
sp|P17625|GYS2_RAT
sp|P13834|GYS1_RABIT
sp|A7MB78|GYS1_BOVIN
S8 (2)
S11 (2a)
C137
C189
C251

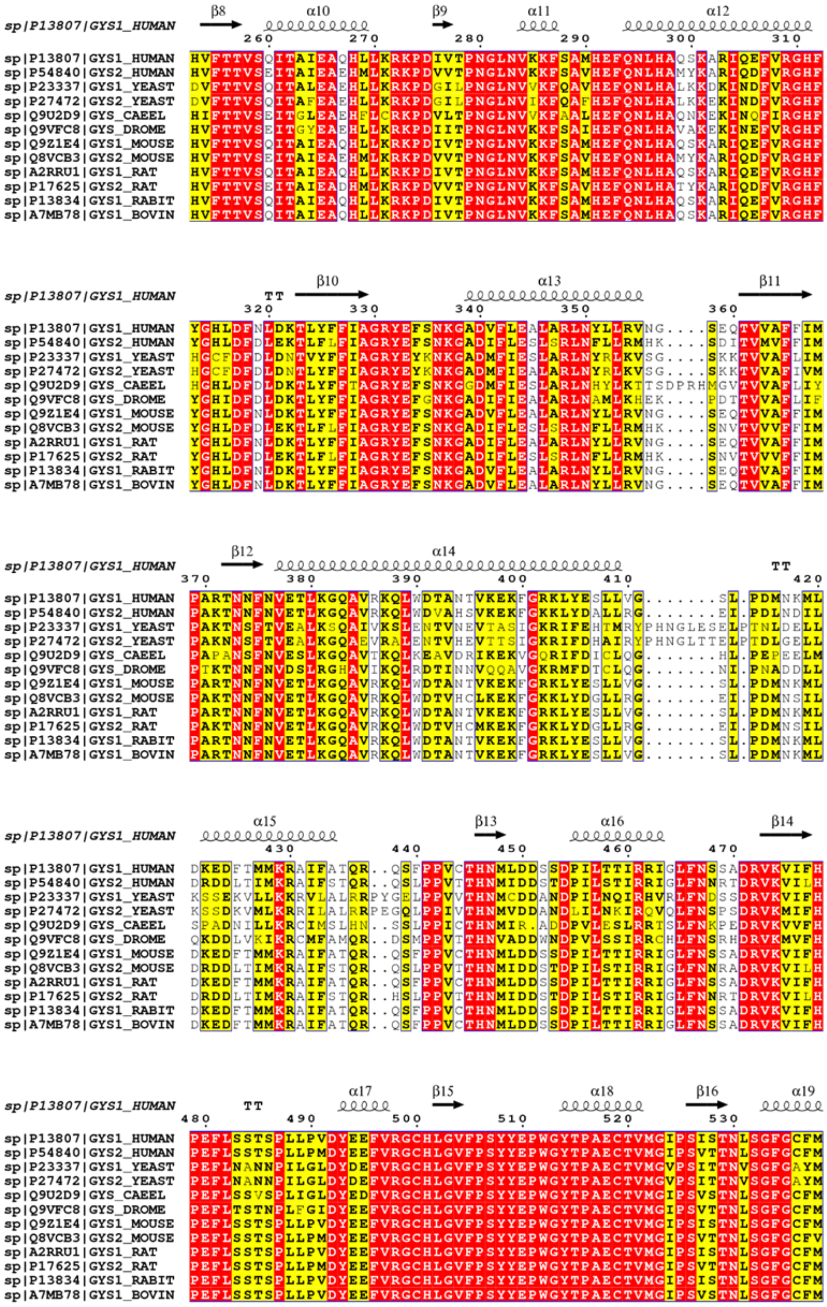
sp|P13807|GYS1_HUMAN
sp|P54840|GYS2_HUMAN
sp|P23337|GYS1_YEAST
sp|P27472|GYS2_YEAST
sp|Q9U2D9|GYS_CAEEL
sp|Q9VFC8|GYS_DROME
sp|Q9Z1E4|GYS1_MOUSE
sp|Q8VCB3|GYS2_MOUSE
sp|A2RRU1|GYS1_RAT
sp|P17625|GYS2_RAT
sp|P13834|GYS1_RABIT
sp|A7MB78|GYS1_BOVIN
β8 α10 β9 α11 α12
β10 α13 β11
β12 α14
α15 β13 α16 β14
α17 β15 α18 β16 α19

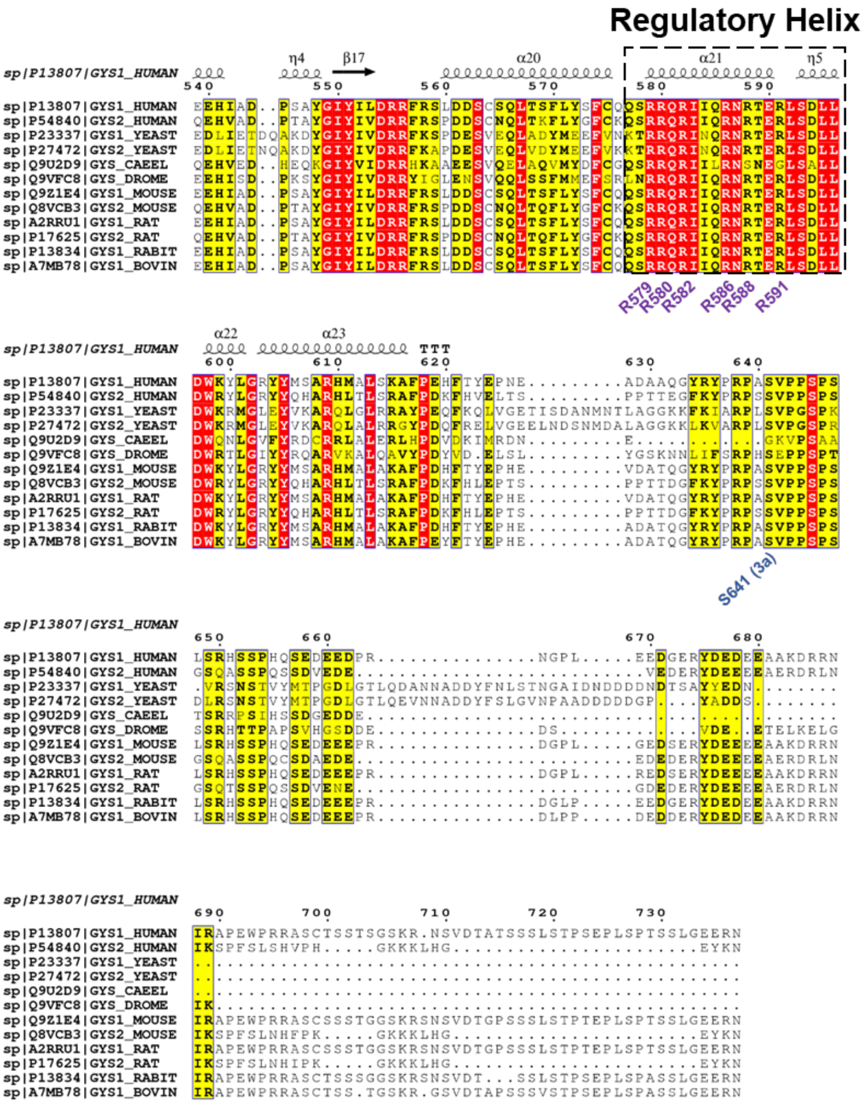

Supplementary Fig. 1 | Sequence alignment of GYS proteins. Key regions and residues are labelled.

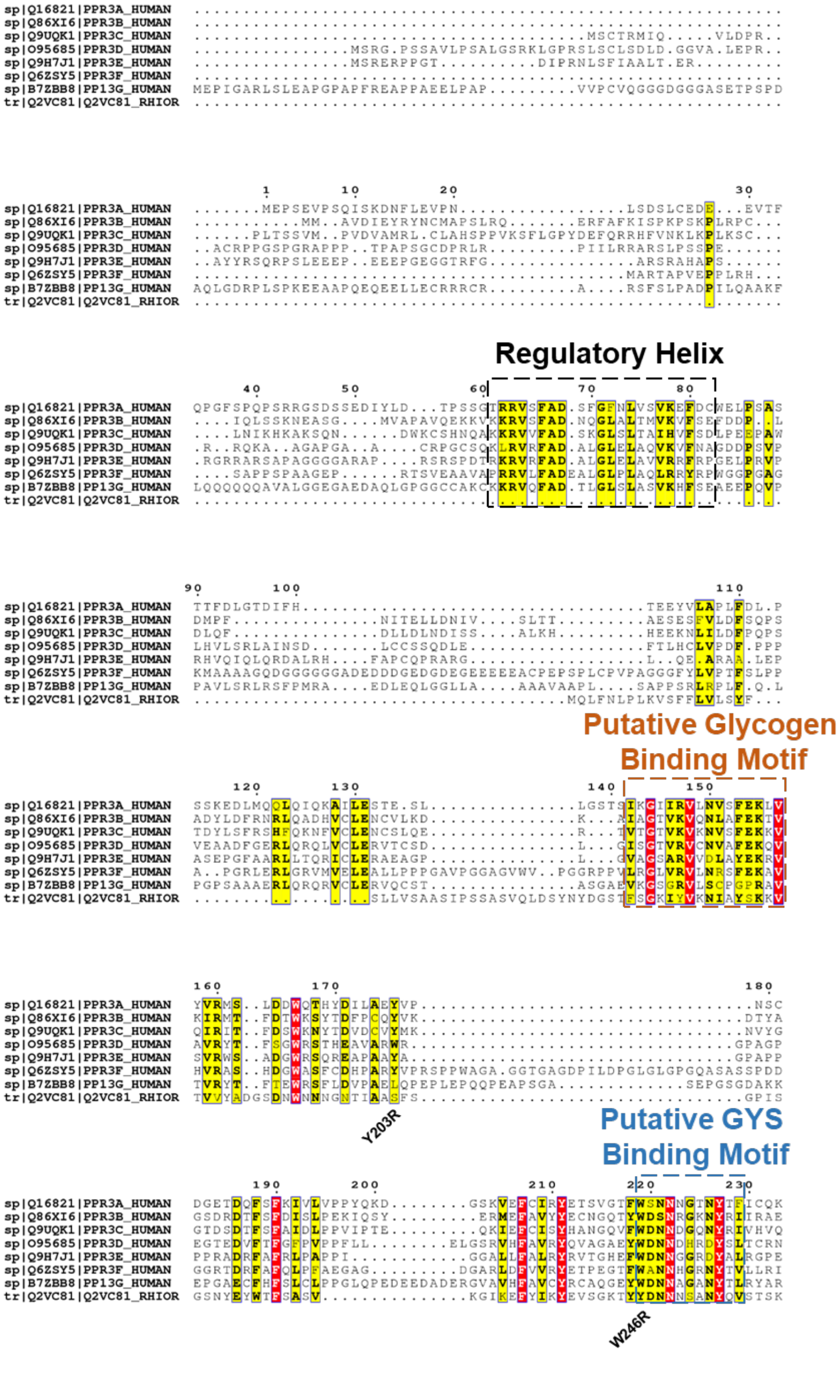

Supplementary Fig. 2 | Sequence alignment of the CBM21 domain of PP1 regulatory subunit PPP1R3 family and *R. oryzae* glucoamylase. Only the N-terminus and CBM21 domain of each PP1 regulatory subunit and *R. oryzae* glucoamylase are shown. Key motifs are highlighted.

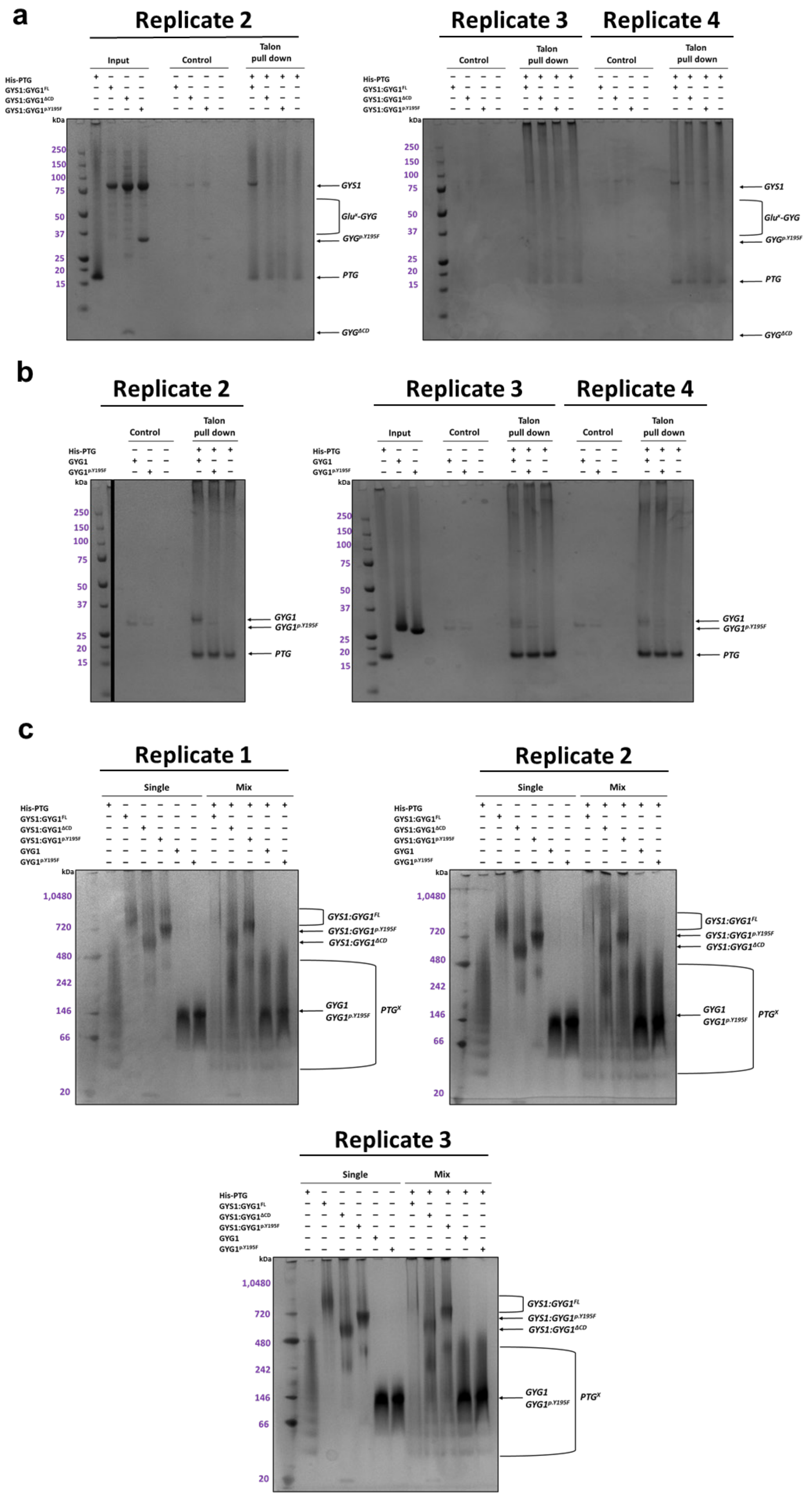
a
Replicate 2
Input
Control
Talon pull down
His-PTG
GYS1:GYG1FL
GYS1:GYG1ΔCD
GYS1:GYG1p.Y195F
kDa
250
150
100
75
50
37
25
20
15
GYS1
Glux-GYG
GYGp.Y195F
PTG
GYGΔCD
Replicate 3
Replicate 4
Control
Talon pull down
b
Replicate 2
Control
Talon pull down
His-PTG
GYG1
GYG1p.Y195F
GYG1
GYG1p.Y195F
PTG
Replicate 3
Replicate 4
Input
Control
Talon pull down
c
Replicate 1
Single
Mix
His-PTG
GYS1:GYG1FL
GYS1:GYG1ΔCD
GYS1:GYG1p.Y195F
GYG1
GYG1p.Y195F
kDa
1,0480
720
480
242
146
66
20
GYS1:GYG1FL
GYS1:GYG1p.Y195F
GYS1:GYG1ΔCD
GYG1
GYG1p.Y195F
PTGX
Replicate 2
Replicate 3

Supplementary Fig. 3 | PTG pulldowns and BN-PAGE replicates. a, Coomassie stained SDS-PAGE replicates of PTG(CBM21) pulldowns against GYS1:GYG1^{FL}, GYS1:GYG1$^{p.Y195F}$, or GYS1:GYG1$^{\Delta CD}$. **b**, Coomassie stained SDS-PAGE replicates of PTG(CBM21) pulldowns against GYG1^{FL} or GYG1$^{p.Y195F}$. **c**, Blue native PAGE shift replicates of PTG(CBM21) incubated with GYS1:GYG1^{FL}, GYS1:GYG1$^{p.Y195F}$, GYS1:GYG1$^{\Delta CD}$, GYG1 alone, or GYG1$^{p.Y195F}$ alone. Complex formation between PTG(CBM21) and GYS1:GYG1^{FL} was inferred from the disappearance of bands compared to other reaction lanes. CBM21 appears as oligomers but behaves as a monomer with some dimer in solution (data not shown).

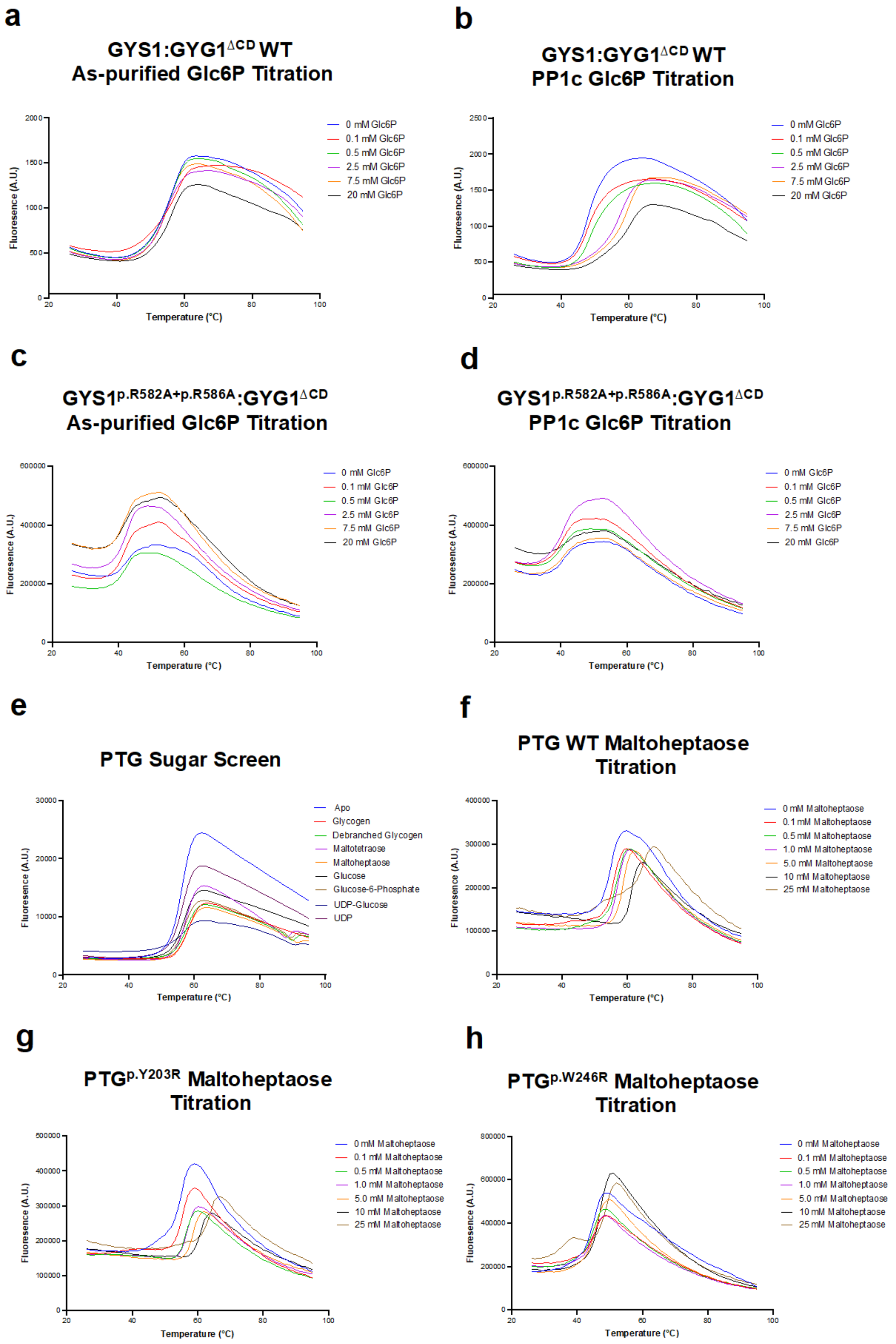
a
GYS1:GYG1$^{\Delta CD}$ WT
As-purified Glc6P Titration
0 mM Glc6P
0.1 mM Glc6P
0.5 mM Glc6P
2.5 mM Glc6P
7.5 mM Glc6P
20 mM Glc6P
Fluoresence (A.U.)
Temperature (°C)
b
GYS1:GYG1$^{\Delta CD}$ WT
PP1c Glc6P Titration
0 mM Glc6P
0.1 mM Glc6P
0.5 mM Glc6P
2.5 mM Glc6P
7.5 mM Glc6P
20 mM Glc6P
Fluoresence (A.U.)
Temperature (°C)
c
GYS1$^{p.R582A+p.R586A}$:GYG1$^{\Delta CD}$
As-purified Glc6P Titration
0 mM Glc6P
0.1 mM Glc6P
0.5 mM Glc6P
2.5 mM Glc6P
7.5 mM Glc6P
20 mM Glc6P
Fluoresence (A.U.)
Temperature (°C)
d
GYS1$^{p.R582A+p.R586A}$:GYG1$^{\Delta CD}$
PP1c Glc6P Titration
0 mM Glc6P
0.1 mM Glc6P
0.5 mM Glc6P
2.5 mM Glc6P
7.5 mM Glc6P
20 mM Glc6P
Fluoresence (A.U.)
Temperature (°C)
e
PTG Sugar Screen
Apo
Glycogen
Debranched Glycogen
Maltotetraose
Maltoheptaose
Glucose
Glucose-6-Phosphate
UDP-Glucose
UDP
Fluoresence (A.U.)
Temperature (°C)
f
PTG WT Maltoheptaose Titration
0 mM Maltoheptaose
0.1 mM Maltoheptaose
0.5 mM Maltoheptaose
1.0 mM Maltoheptaose
5.0 mM Maltoheptaose
10 mM Maltoheptaose
25 mM Maltoheptaose
Fluoresence (A.U.)
Temperature (°C)
g
PTG$^{p.Y203R}$ Maltoheptaose Titration
0 mM Maltoheptaose
0.1 mM Maltoheptaose
0.5 mM Maltoheptaose
1.0 mM Maltoheptaose
5.0 mM Maltoheptaose
10 mM Maltoheptaose
25 mM Maltoheptaose
Fluoresence (A.U.)
Temperature (°C)
h
PTG$^{p.W246R}$ Maltoheptaose Titration
0 mM Maltoheptaose
0.1 mM Maltoheptaose
0.5 mM Maltoheptaose
1.0 mM Maltoheptaose
5.0 mM Maltoheptaose
10 mM Maltoheptaose
25 mM Maltoheptaose
Fluoresence (A.U.)
Temperature (°C)

Supplementary Fig. 4 | Representative thermal shift unfolding curves of each GYS1:GYG1 complex and PTG(CBM21) mutant. **a**, GYS1:GYG1$^{\Delta CD}$ WT as-purified, titrated with increasing Glc6P. **b**, GYS1:GYG1$^{\Delta CD}$ WT + PP1c, titrated with increasing Glc6P. **c**, GYS1$^{p.R582A+p.R586A}$:GYG1$^{\Delta CD}$ as-purified, titrated with increasing Glc6P. **d**, GYS1$^{p.R582A+p.R586A}$:GYG1$^{\Delta CD}$ + PP1c, titrated with increasing Glc6P. **e**, PTG(CBM21) screened against various sugars and ligands at 1 mM each. **f**, PTG(CBM21) WT, titrated with increasing maltoheptaose. **g**, PTG(CBM21)$^{p.Y203R}$, titrated with increasing maltoheptaose. **h**, PTG(CBM21)$^{p.W246R}$, titrated with increasing maltoheptaose.

Supplementary Table 1 | Oligonucleotide sequences used for mutagenesis

Primer	Sequence
GYS1_R582A+R586A_Fr	GGCCATCATCCAGGCCAACCGCACGGAGCGCCTC
GYS1_R582A+R586A_Rv	GCCTGGATGATGGCCTGCCGCCGGCTCTGCTG
PTG_W246R_Fr	AGTCTTTCGGGACAACAATGATGGTCAGAATT
PTG_W246R_Rv	TTGTCCCGAAAGACTTGCCCATTAGCA
PTG_Y203R_Fr	CTGTGTCCGTATGAAAAATGTGTATGGTGGCACAG
PTG_Y203R_Rv	TTCATACGGACACAGTCTACGTCAGTGTAG

Uncropped image of Supplementary Fig. 3a

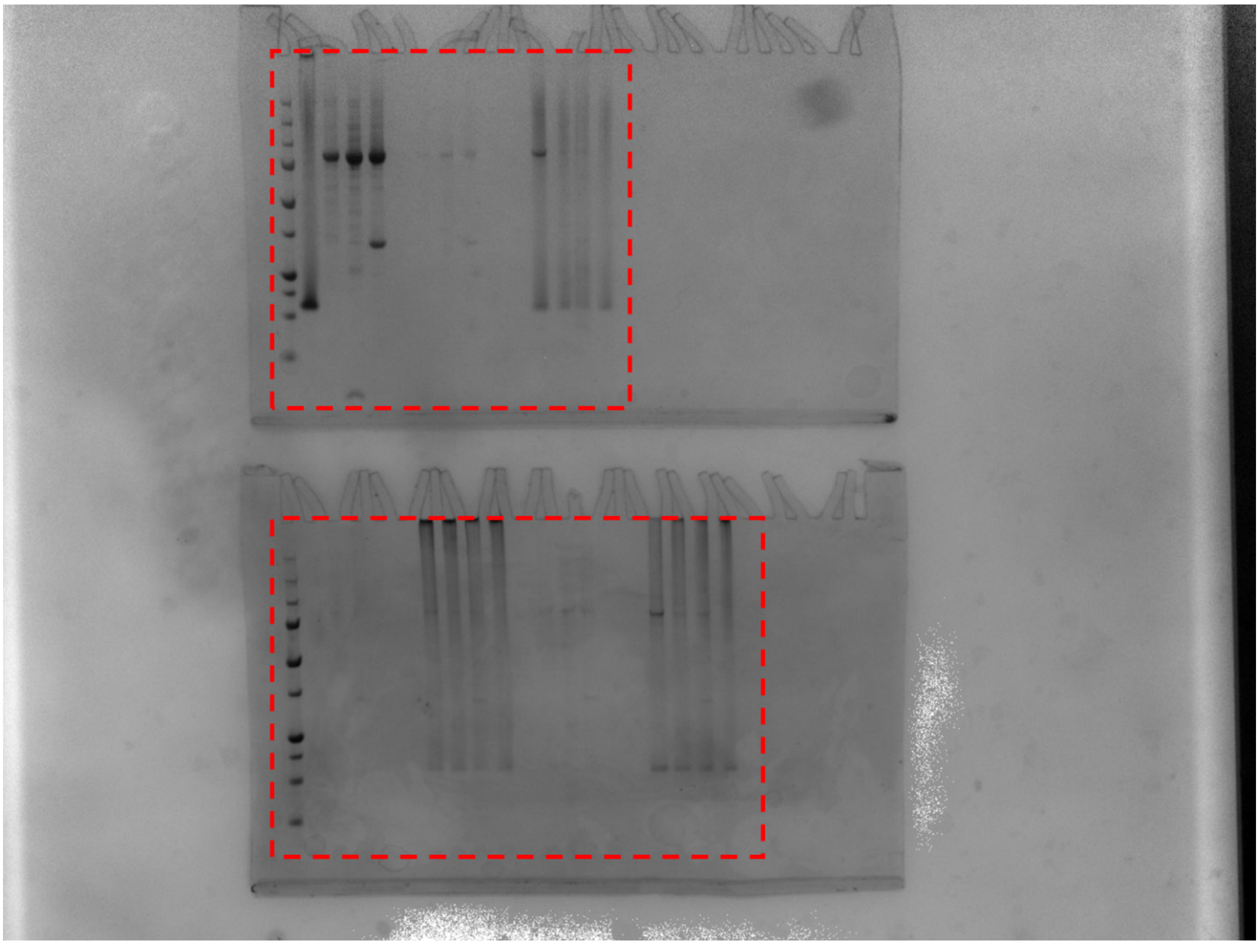

Uncropped image of Supplementary Fig. 3b

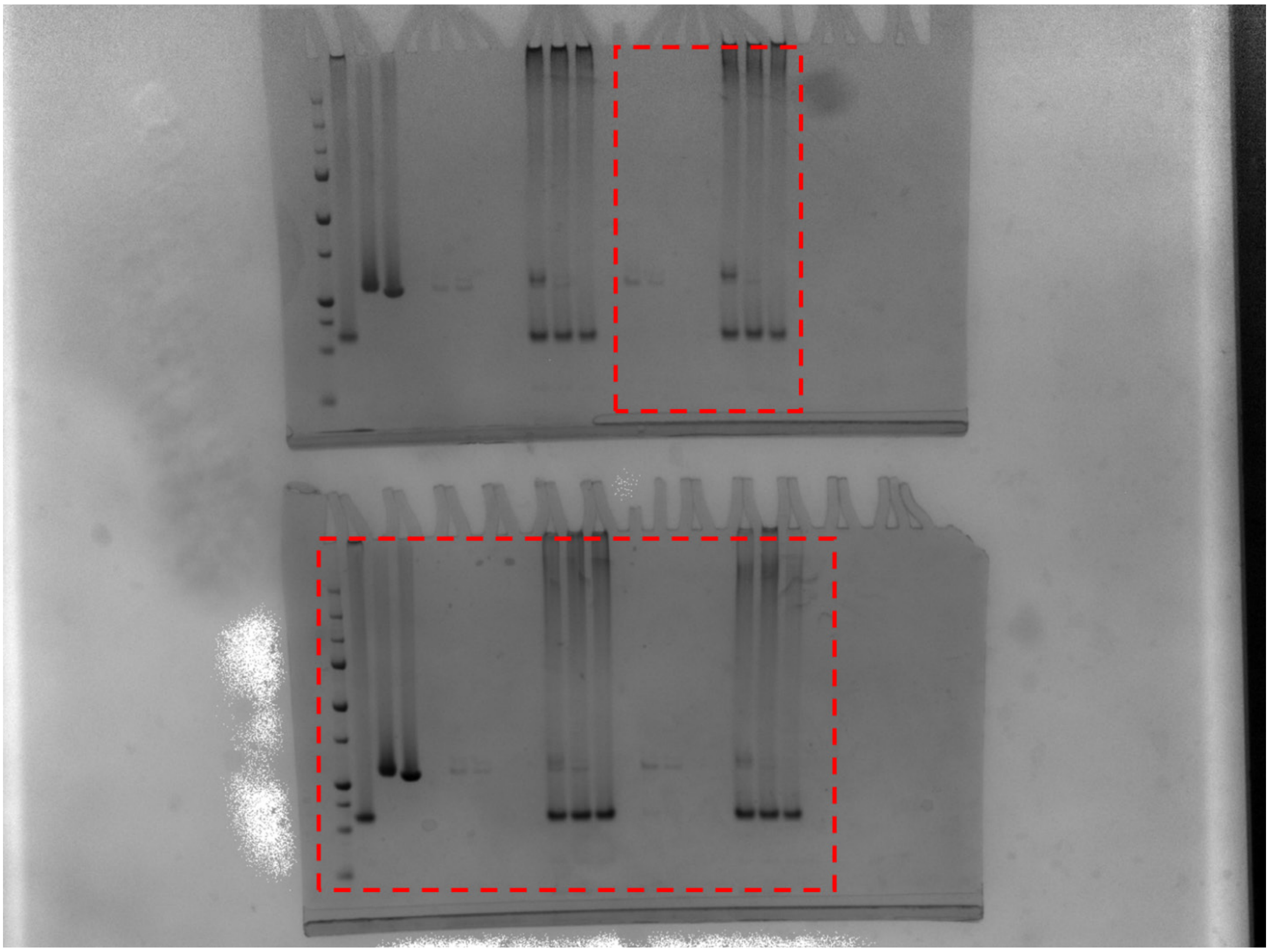

Uncropped image of Supplementary Fig. 3c

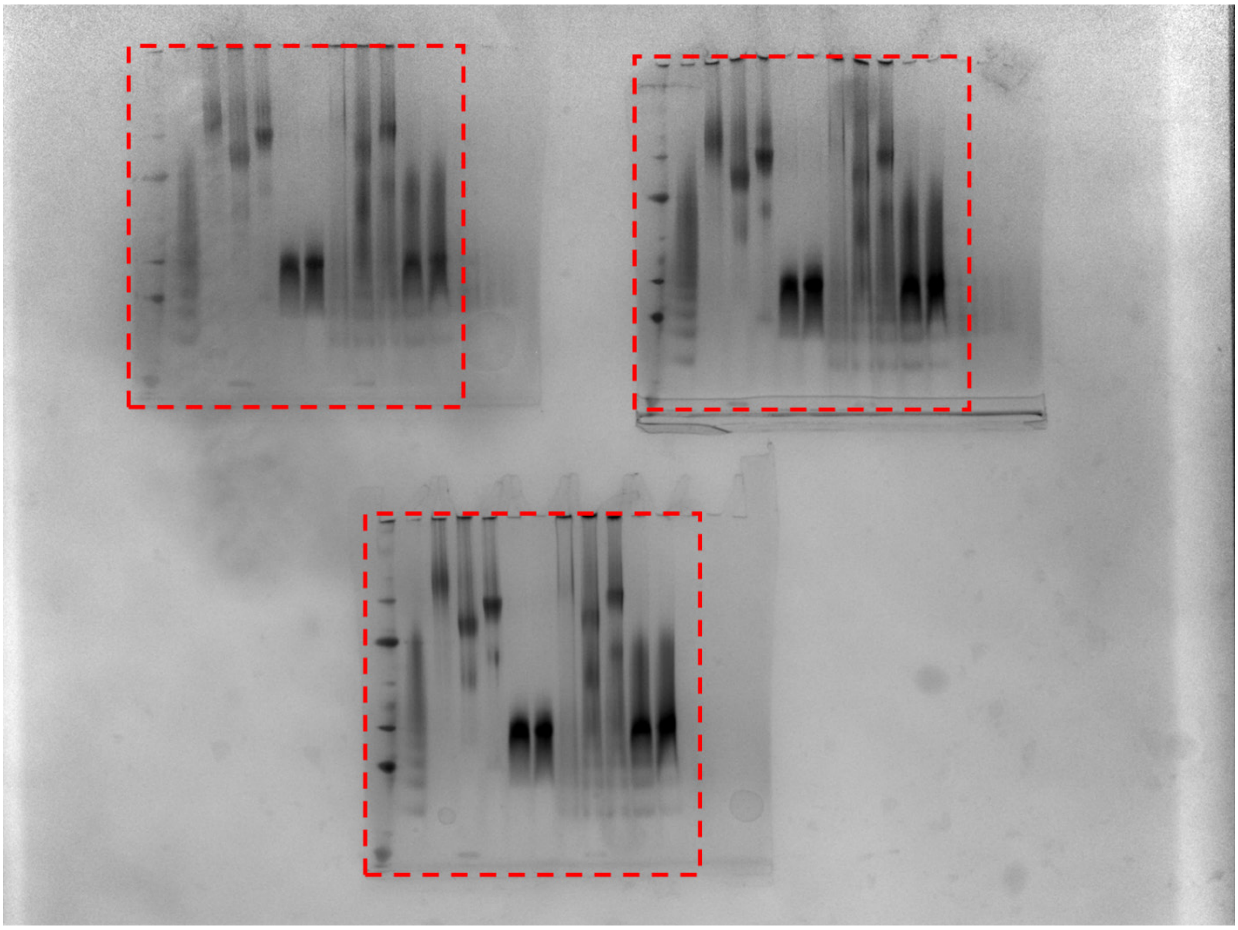

Supplement: Supplementary file 1 — Supplementary Figs. 1–4, Supplementary Table 1, uncropped image for Supplementary Fig. 3a, uncropped gel image for Supplementary Fig. 3b, uncropped gel image for Supplementary Fig. 3c. [file 41594_2022_799_MOESM1_ESM.pdf]
