# Supplementary material for: Molecular basis for the regulation of human glycogen synthase by phosphorylation and glucose-6-phosphate
Source: Nat Struct Mol Biol. 2022 Jul 14;29(7):628–38. doi: 10.1038/s41594-022-00799-3 (PMC9287172; doi:10.1038/s41594-022-00799-3)
Supplement: Supplementary file 2 — Reporting Summary [file 41594_2022_799_MOESM2_ESM.pdf]

nature portfolio

Corresponding author(s): Allison P. Berg, Wyatt Yue

Last updated by author(s): Apr 25, 2022

Reporting Summary

Nature Portfolio wishes to improve the reproducibility of the work that we publish. This form provides structure for consistency and transparency in reporting. For further information on Nature Portfolio policies, see our Editorial Policies and the Editorial Policy Checklist.

Statistics

For all statistical analyses, confirm that the following items are present in the figure legend, table legend, main text, or Methods section.

n/a	Confirmed	
☐	☒	The exact sample size (*n*) for each experimental group/condition, given as a discrete number and unit of measurement
☐	☒	A statement on whether measurements were taken from distinct samples or whether the same sample was measured repeatedly
☐	☒	The statistical test(s) used AND whether they are one- or two-sided<br>*Only common tests should be described solely by name; describe more complex techniques in the Methods section.*
☒	☐	A description of all covariates tested
☒	☐	A description of any assumptions or corrections, such as tests of normality and adjustment for multiple comparisons
☐	☒	A full description of the statistical parameters including central tendency (e.g. means) or other basic estimates (e.g. regression coefficient) AND variation (e.g. standard deviation) or associated estimates of uncertainty (e.g. confidence intervals)
☐	☒	For null hypothesis testing, the test statistic (e.g. *F*, *t*, *r*) with confidence intervals, effect sizes, degrees of freedom and *P* value noted<br>*Give P values as exact values whenever suitable.*
☒	☐	For Bayesian analysis, information on the choice of priors and Markov chain Monte Carlo settings
☒	☐	For hierarchical and complex designs, identification of the appropriate level for tests and full reporting of outcomes
☒	☐	Estimates of effect sizes (e.g. Cohen's *d*, Pearson's *r*), indicating how they were calculated

Our web collection on statistics for biologists contains articles on many of the points above.

Software and code

Policy information about availability of computer code

Data collection	DLS beamline Krios, Midlands Regional CryoEM Facility Krios, SpectraMax M3 (Molecular Devices), Mx3005p RT-PCR machine (Stratagene)
Data analysis	RELION v3.1, CryoSPARC v3.1, GraphPad Prism v9.3.1, COOT v0.9.7, SWISS-MODEL, MotionCor2 v1.4.0, CTFfind4 v4.1.9, UCSF Chimera v1.3, PHENIX v1.19.2-4158, Namdinator

For manuscripts utilizing custom algorithms or software that are central to the research but not yet described in published literature, software must be made available to editors and reviewers. We strongly encourage code deposition in a community repository (e.g. GitHub). See the Nature Portfolio guidelines for submitting code & software for further information.

Data

Policy information about availability of data

All manuscripts must include a data availability statement. This statement should provide the following information, where applicable:

- Accession codes, unique identifiers, or web links for publicly available datasets
- A description of any restrictions on data availability
- For clinical datasets or third party data, please ensure that the statement adheres to our policy

Datasets generated during the current study are available from the Protein Data Bank (PDB) accession codes 7Q0B, 7Q0S, 7Q12 and 7Q13, and Electron Microscopy Data Bank (EMDB) accession codes 13743, 13751, 13752, and 13753. All main data supporting the findings of this study are available within the article, Extended Data, and Supplementary Information. Source data are provided with this paper. Other data are available from the corresponding author upon reasonable request.

Field-specific reporting

Please select the one below that is the best fit for your research. If you are not sure, read the appropriate sections before making your selection.

☒ Life sciences ☐ Behavioural & social sciences ☐ Ecological, evolutionary & environmental sciences

For a reference copy of the document with all sections, see nature.com/documents/nr-reporting-summary-flat.pdf

Life sciences study design

All studies must disclose on these points even when the disclosure is negative.

Sample size	No sample size calculation performed. For cryoEM the sample size was determined by the ability to reach an overall resolution better than or equal to 4.0 Å to ensure confidence in conclusions drawn from the data.
Data exclusions	No data excluded.
Replication	Activity measurements, pull-downs and native PAGE experiments were carried out in technical replicates of N=3 or 4 as stated in figure legends
Randomization	Not applicable - no experimental groups were involved.
Blinding	Not applicable - no group allocation was involved.

Reporting for specific materials, systems and methods

We require information from authors about some types of materials, experimental systems and methods used in many studies. Here, indicate whether each material, system or method listed is relevant to your study. If you are not sure if a list item applies to your research, read the appropriate section before selecting a response.

Materials & experimental systems

n/a	Involved in the study	
☒	☐	Antibodies
☐	☒	Eukaryotic cell lines
☒	☐	Palaeontology and archaeology
☒	☐	Animals and other organisms
☒	☐	Human research participants
☒	☐	Clinical data
☒	☐	Dual use research of concern

Methods

n/a	Involved in the study	
☒	☐	ChIP-seq
☒	☐	Flow cytometry
☒	☐	MRI-based neuroimaging

Eukaryotic cell lines

Policy information about cell lines

Cell line source(s)	Spodoptera frugiperda (Thermo-Fisher Scientific, cat# 11496015)
Authentication	No cells lines used were authenticated
Mycoplasma contamination	Cell lines were not tested for mycoplasma contamination
Commonly misidentified lines (See ICLAC register)	Not applicable to Sf9 cultures
