# Supplementary figures and images for: Molecular basis for the regulation of human glycogen synthase by phosphorylation and glucose-6-phosphate
Source: Nat Struct Mol Biol. 2022 Jul 14;29(7):628–38. doi: 10.1038/s41594-022-00799-3 (PMC9287172; doi:10.1038/s41594-022-00799-3)

**Uncropped image of Fig. 6b**

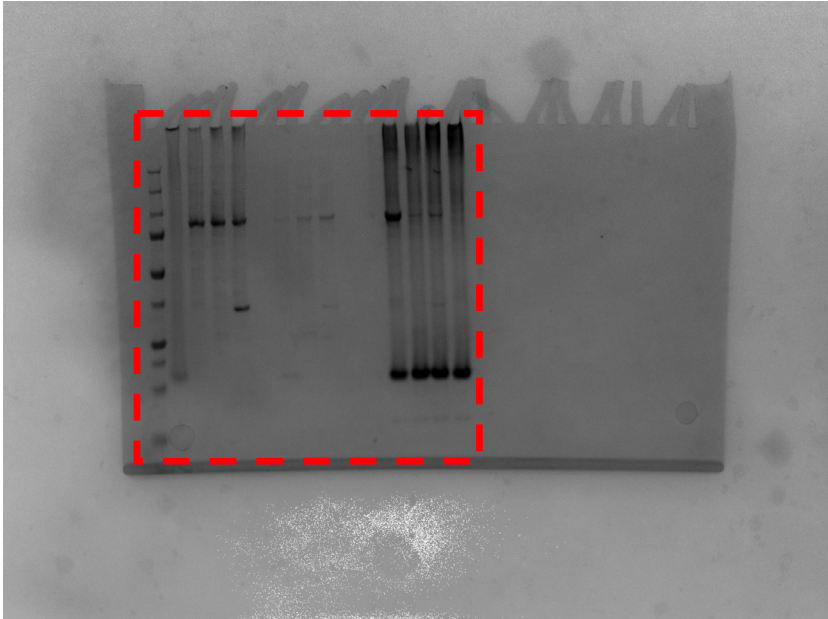

**Uncropped image of Fig. 6C**

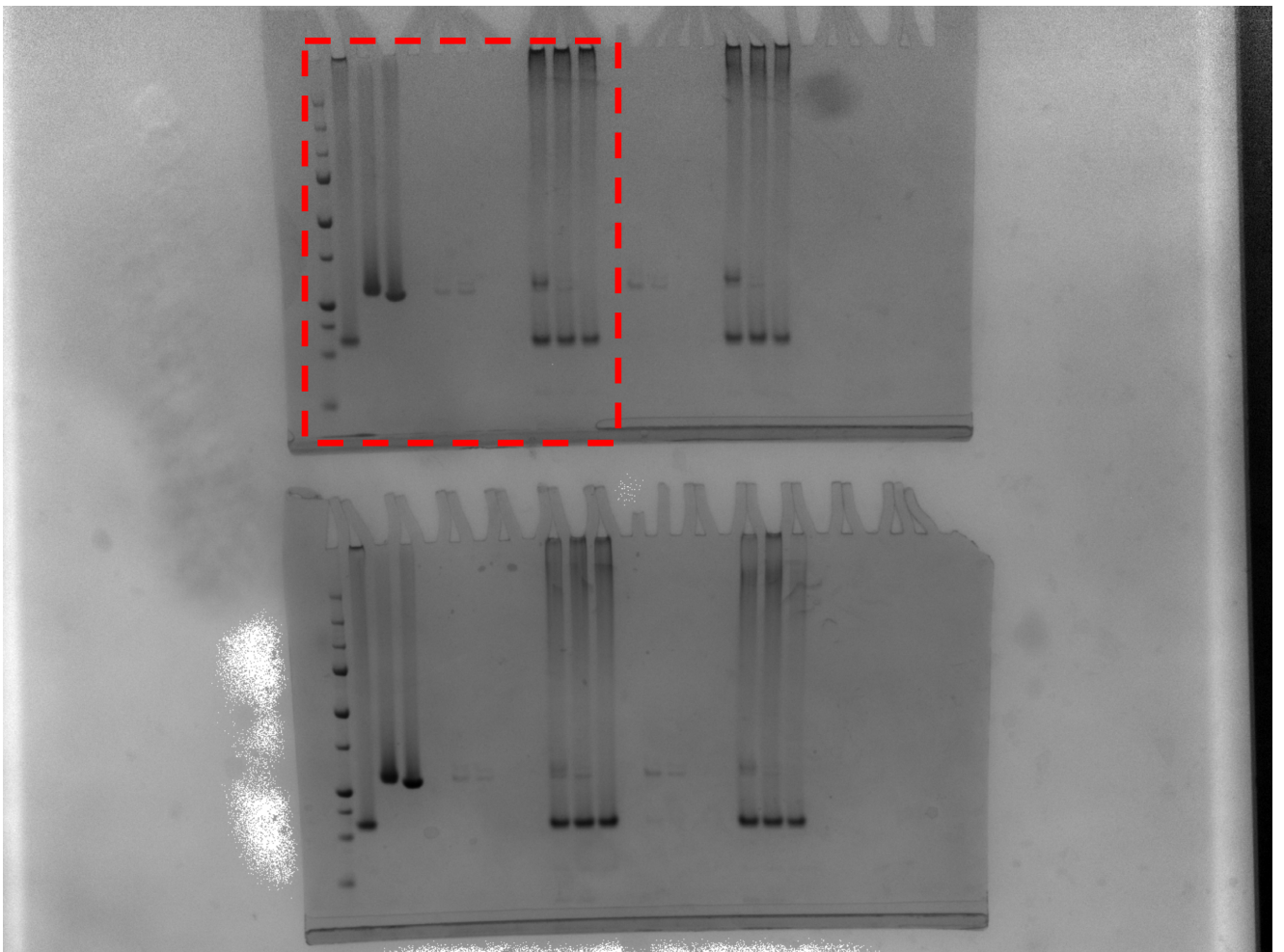

Supplement: Source Data Fig. 6 — Uncropped gels for Fig. 6b,c. [file 41594_2022_799_MOESM10_ESM.pdf]

Uncropped image of Extended Data Fig.8a

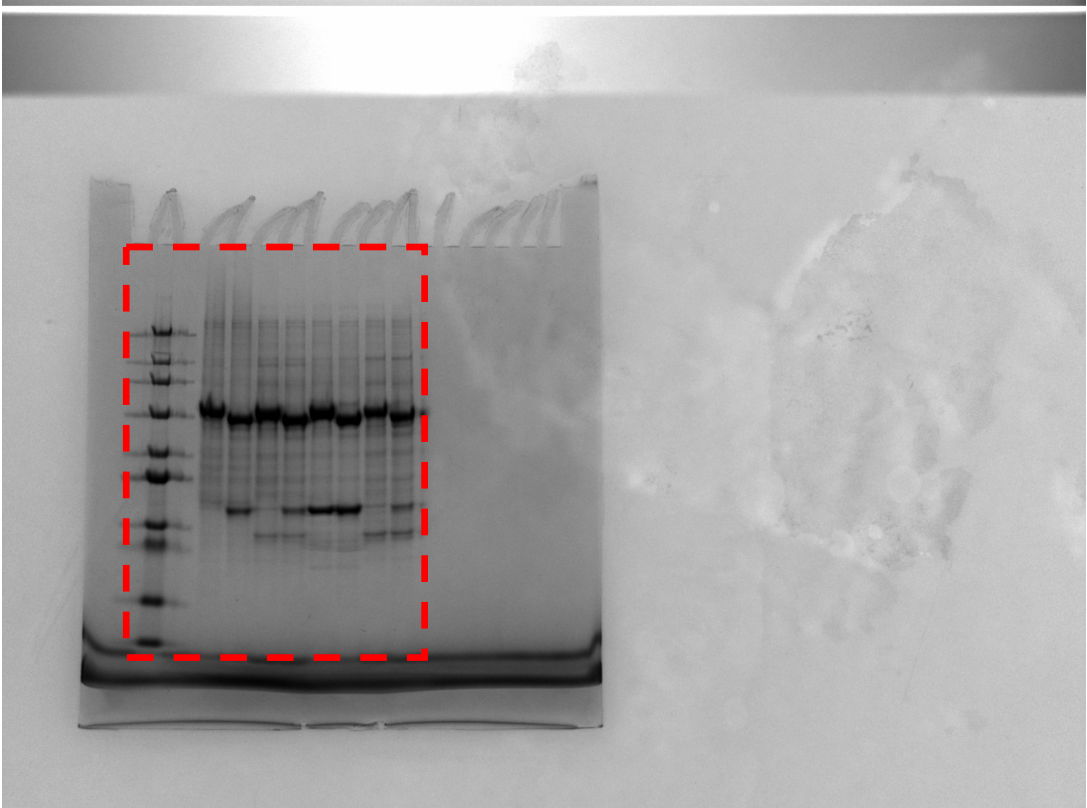

Supplement: Source Data Extended Data Fig. 8 — Uncropped gel for Extended Data Fig. 8a. [file 41594_2022_799_MOESM14_ESM.pdf]
