# Supplementary material for: Molecular basis for the regulation of human glycogen synthase by phosphorylation and glucose-6-phosphate
Source: Nat Struct Mol Biol. 2022 Jul 14;29(7):628–38. doi: 10.1038/s41594-022-00799-3 (PMC9287172; doi:10.1038/s41594-022-00799-3)

Uncropped image of Extended Data Fig. 1a

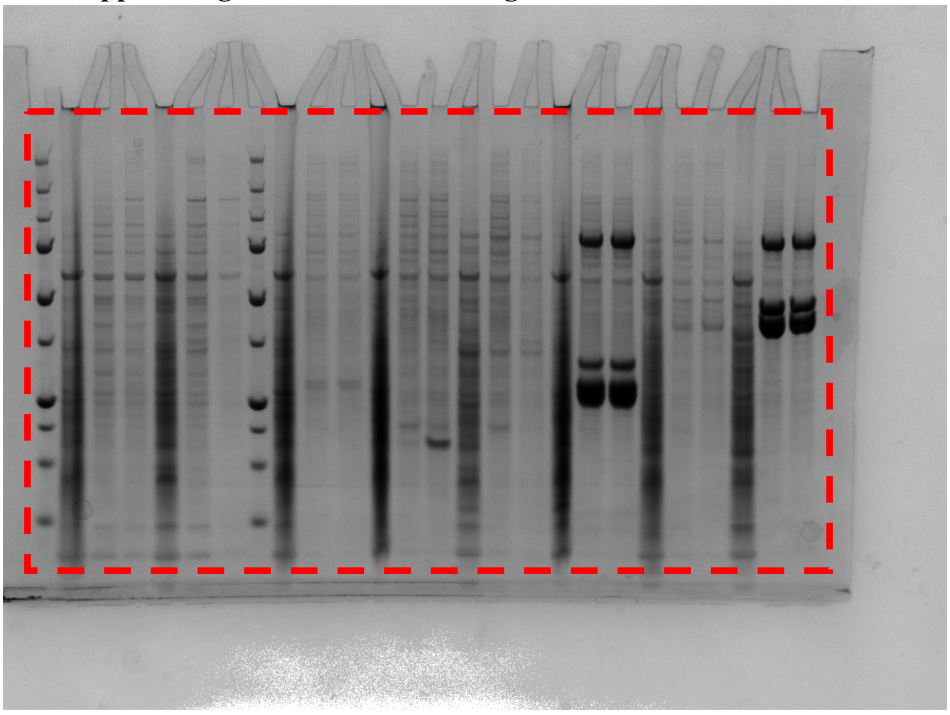

Uncropped image of Extended Data Fig. 1b

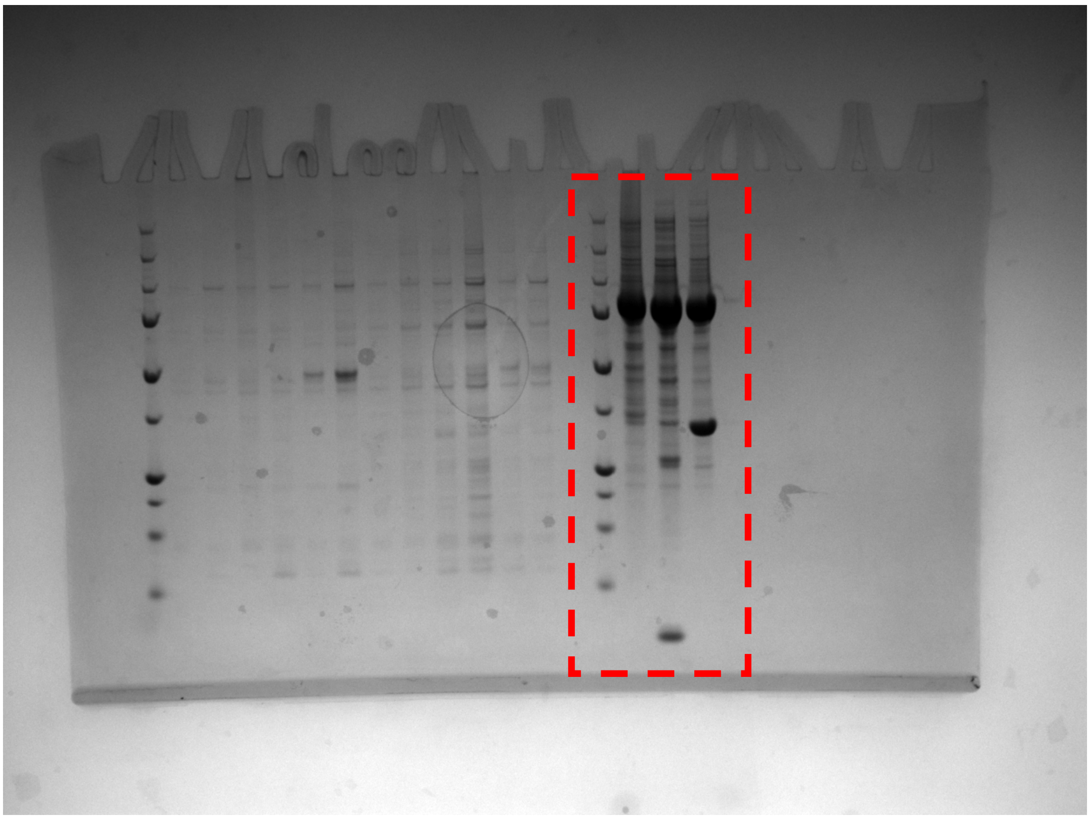

Uncropped image of Extended Data Fig. 1c

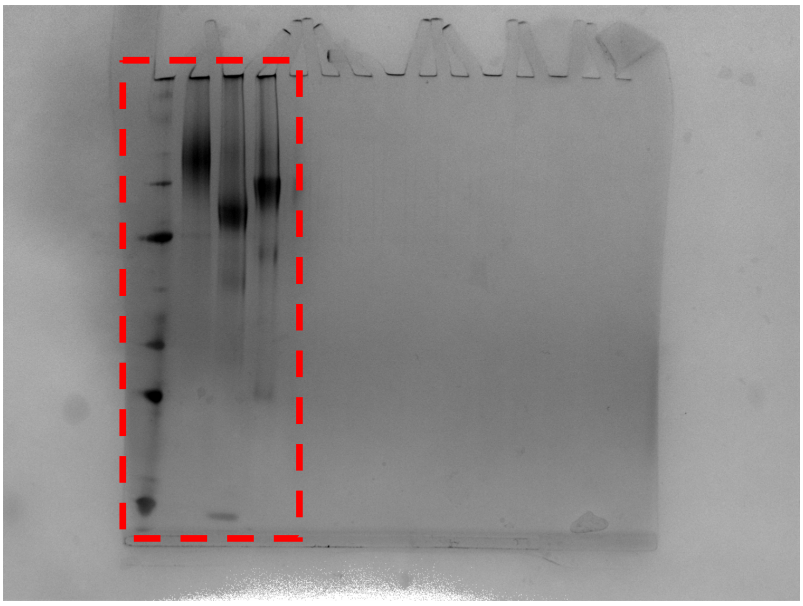

Supplement: Source Data Extended Data Fig. 1 — Uncropped gels for Extended Data Fig. 1a–c. [file 41594_2022_799_MOESM12_ESM.pdf]
